# Graph attention network‐driven single‐cell omics analysis reveals how B4GALNT1‐mediated glycosylation remodelling of the ferroptosis–pyroptosis network promotes drug resistance in hepatocellular carcinoma

**DOI:** 10.1002/ctm2.70873

**Published:** 2026-10-15

**Authors:** Lihan Bie, Biao Xu, Haitao Shen, Shuwen Ren, Xiaoyang Yao, Zhouhong Xiang, Zheng Xu

**Affiliations:** ^1^ Department of Laboratory Medicine The Seventh People's Hospital Affiliated to Shanghai University of Traditional Chinese Medicine Shanghai China

**Keywords:** B4GALNT1, ferroptosis, ganglioside, glycosylation, hepatocellular carcinoma, multi‐omics, pyroptosis, sorafenib resistance

## Abstract

**Background:**

Acquired resistance to sorafenib in hepatocellular carcinoma (HCC) is accompanied by coordinated changes in oxidative‐stress handling and regulated cell‐death pathways, yet how membrane organisation connects these adaptations is not fully resolved. We therefore examined whether B4GALNT1, a ganglioside biosynthetic glycosyltransferase, couples changes in ganglioside composition and lipid‐raft architecture to ferroptosis regulation and caspase‐1/GSDMD‐associated signalling in sorafenib‐resistant HCC.

**Methods:**

We combined TCGA‐LIHC analysis, single‐cell transcriptomics, pathway‐level analyses, and graph attention network modelling with a treatment‐annotated local cohort of 92 patients with HCC who received sorafenib. Functional experiments were conducted in Huh7/Huh7‐SR cells and additional liver‐tumour models using B4GALNT1 gain‐ and loss‐of‐function, an independent second‐sgRNA knockout clone, GM1 rescue, and re‐expression of either wild‐type B4GALNT1 or the catalytic‐loss D358A variant. Validation included ganglioside profiling, detergent‐resistant membrane isolation, lipid‐raft disruption/repletion, ferroptosis‐directed pharmacology, cell‐death measurements, and xenograft studies.

**Results:**

B4GALNT1 was enriched in tumour‐associated hepatocyte populations and was upregulated in Huh7‐SR cells. In the local cohort, higher B4GALNT1 protein abundance tracked with less favourable response to sorafenib and poorer survival outcomes. Increasing B4GALNT1 shifted ganglioside abundance toward GM2/GM1/GD2 and increased GM1‐enriched raft‐associated fractions, whereas knockout produced reciprocal changes. B4GALNT1‐high cells showed increased GPX4 and SLC7A11, reduced lipid peroxidation and labile Fe^2^^+^, and lower ferroptotic injury. At the same time, caspase‐1/GSDMD‐associated signalling and IL‐1β/IL‐18 release increased without widespread terminal membrane lysis. Exogenous GM1 partially rescued knockout‐associated changes, and wild‐type B4GALNT1 restored the phenotype more effectively than the D358A mutant. Concordant directional effects were observed in additional cell models and in xenografts.

**Conclusions:**

The data are consistent with a model in which B4GALNT1 reshapes ganglioside composition and GM1‐enriched membrane microdomains, thereby supporting a drug‐adapted state that combines greater ferroptosis resistance with restricted caspase‐1/GSDMD‐associated membrane permeabilisation. This integrated phenotype accompanies reduced sorafenib responsiveness and offers a mechanistic basis for acquired resistance in HCC.

**HIGHLIGHTS:**

- B4GALNT1 is associated with sorafenib resistance and poorer outcomes in treated HCC.
- B4GALNT1 remodels gangliosides and lipid rafts, limiting ferroptotic injury.
- Caspase‐1/GSDMD signalling increases without widespread terminal membrane lysis.
- Genetic rescue and cross‐model studies support B4GALNT1 specificity.

## Graphical abstract

**Summary: B4GALNT1‐driven ganglioside/raft remodelling links ferroptosis suppression with restricted caspase‐1/GSDMD signalling during sorafenib resistance**.

Note: Schematic summary of the proposed B4GALNT1–ganglioside–lipid‐raft axis linking ferroptosis protection, restricted caspase‐1/GSDMD‐associated membrane permeabilisation, and sorafenib resistance.

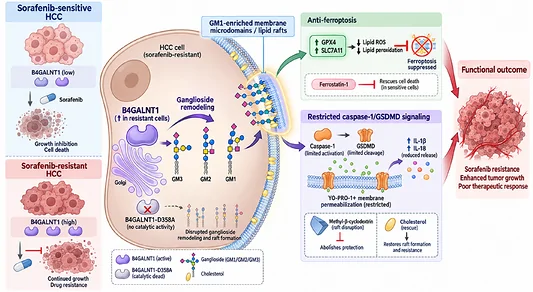

## 1 BACKGROUND

Hepatocellular carcinoma (HCC) contributes substantially to global cancer mortality, with liver cancer showing marked geographic variation in both incidence and death rates according to GLOBOCAN 2022.^1^, ^2^ For advanced or unresectable disease, systemic treatment remains central to disease control. Sorafenib, a multikinase inhibitor, has long been used in advanced HCC and has produced an overall‐survival benefit in randomised studies.^3^ Its clinical activity, however, is limited by modest response rates and the frequent emergence of acquired resistance during prolonged exposure. Loss of drug sensitivity shortens the duration of benefit and facilitates subsequent progression. Mechanistically, sorafenib resistance is multifactorial and has been linked to altered drug handling, compensatory signalling, microenvironmental adaptation, and rewiring of regulated cell‐death pathways.^4^, ^5^ A better understanding of upstream regulators that coordinate several of these adaptive processes may reveal how resistant states are established and maintained.

Drug‐exposed tumour cells can survive by resetting the thresholds that govern regulated cell death. Ferroptosis is defined by iron‐dependent accumulation of damaging lipid peroxides and has become an important component of the sorafenib‐resistance framework in HCC.^6^, ^7^, ^8^ Pyroptosis, in contrast, is an inflammatory membrane‐permeabilising program in which inflammatory caspases cleave gasdermins such as GSDMD to generate pore‐forming fragments.^9^ Structural and biochemical work has clarified how CASP1 recognises and processes GSDMD, establishing a molecular basis for this execution step.^10^ In cancer, however, the consequences of pyroptotic signalling can vary with context and signal intensity, and several reports discuss a continuum between fully lytic and sublethal states.^11^, ^12^, ^13^ These observations raise the possibility that drug resistance may involve coordinated adjustment of ferroptotic defence, inflammatory‐caspase activity, and the extent of membrane permeabilisation rather than isolated changes in a single death pathway.

Cancer‐associated glycosylation changes can influence receptor trafficking, cell surface interactions, metabolism, and signal organisation, thereby contributing to tumour progression and treatment adaptation.^14^, ^15^, ^16^ Membrane glycosphingolipids add another regulatory layer because their composition affects the physical and signalling properties of the plasma membrane. Cholesterol‐ and sphingolipid‐rich lipid rafts act as dynamic membrane domains that concentrate selected receptors and signalling proteins and can alter pathway efficiency or partner availability.^17^, ^18^ Gangliosides are prominent raft‐associated glycosphingolipids, and GM1 in particular can modulate receptor organisation within these domains.^19^, ^20^ In liver cancer models, interference with ganglioside biosynthesis has been reported to reduce proliferation and spheroid growth,^21^ while broader reviews of HCC highlight potential links among glycosphingolipids, stem‐like traits, and therapy resistance.^22^ On this basis, altered ganglioside synthesis may influence drug adaptation by reshaping membrane microdomains that control stress and cell‐death signalling.

## 2 MATERIALS AND METHODS

### 2.1 Public data acquisition, differential expression analysis, and bioinformatics analysis

TCGA‐LIHC transcriptomic data were retrieved from the Genomic Data Commons portal (https://portal.gdc.cancer.gov/). The expression dataset contained 374 tumour/adjacent non‐tumour specimens, and clinical annotations were available for 377 patients, including follow‐up time (futime), survival status (fustat), stage, and T stage. Because these records are publicly accessible and de‐identified, no additional ethics approval or informed consent was required for this component of the study.

Differential‐expression testing was carried out in R with limma. Raw *p* values were corrected for multiple testing by false‐discovery‐rate adjustment, and genes with FDR < .05 were retained as differentially expressed. The resulting expression changes were displayed as volcano plots generated with ggplot2.

For GSEA, samples were divided at the median B4GALNT1 expression into high‐ and low‐expression strata and analysed with clusterProfiler in R. Enrichment plots were produced with enrichplot, and gene sets meeting FDR < .05 were considered significant.

Clinical‐association analyses paired the expression matrix with survival information. Patients were dichotomised by the median B4GALNT1 level, after which survival curves were generated with the R survival package. Discrimination between tumour and non‐tumour samples was additionally assessed by ROC analysis using pROC.

B4GALNT1 expression was compared across T, N, M, and AJCC stage categories (Stage I–II versus Stage III–IV) with the Kruskal–Wallis test implemented in R, followed by Dunn post hoc comparisons where appropriate. Plots were prepared with ggpubr (v0.4.0) and ggplot2.

Expression values for B4GALNT1, GSDMD, SLC7A11, CASP1, and GCNT2 were extracted from the TCGA‐LIHC matrix for correlation analysis. Pairwise associations were evaluated by Spearman's rank correlation within the same cohort, and scatter plots were annotated with the corresponding correlation coefficients and *p* values.

### 2.2 Acquisition and analysis of single‐cell RNA sequencing (scRNA‐seq) data

Single‐cell transcriptomic analysis used GEO dataset GSE202642 (https://www.ncbi.nlm.nih.gov/geo/). Three adjacent‐normal samples (GSM6127499, GSM6127500, GSM6127501) and three tumour samples (GSM6127506, GSM6127507, GSM6127508) were selected. These publicly available, de‐identified data did not require new ethics approval or informed consent.

Cell identities were assigned from established marker‐gene patterns with support from the CellMarker database. For each specimen, the relative abundance of annotated cell types was calculated and compared between normal and tumour sources. FeaturePlot was used to map B4GALNT1, GPX4, GSDMD, and IL1B expression within the single‐cell atlas.

Hepatocytes were isolated computationally and subjected to a second round of clustering with the same processing workflow to resolve transcriptionally distinct subgroups. Predefined gene sets were used to derive anti‐ferroptosis and pyroptosis‐susceptibility scores, which were then compared between normal and tumour hepatocytes. Within the hepatocyte compartment, B4GALNT1, GPX4, GSDMD, and IL1B expression values were also used for correlation analyses and scatter‐plot visualisation.

### 2.3 Graph attention network (GAT) model construction and key gene network analysis

SVM and RF classifiers were implemented alongside the GAT model to provide method‐level comparators. Each algorithm received the same expression features and used the same train/test partition. Performance was summarised by ROC analysis and AUC, and classification outcomes were further inspected with confusion matrices.

Candidate‐gene importance values output by the GAT were normalised and ranked. The relative positions of B4GALNT1 and other high‐priority genes were then visualised for downstream interpretation.

For network visualisation, the candidate set and model‐derived relationships were organised into glycosylation, ferroptosis, pyroptosis, and bridge modules. Connections were taken from the modelled association structure and the predefined graph topology, and inter‐module relationships were displayed with Circos plots.

### 2.4 Cell culture and grouping

Huh7 cells (CL‐0120, Procell, Wuhan, China) were cultured at 37°C/5% CO_2_ in high‐glucose DMEM (Thermo Fisher Scientific, USA) containing 10% FBS (Gibco, USA) and 1% penicillin/streptomycin (100 U/mL; Gibco, USA). Group labels reflected the construct or intervention used in each experiment: parental cells were denoted WT when applicable, empty‐vector controls were labelled Vector or Oe‐NC, and manipulated groups were designated Oe‐B4GALNT1 or KO‐B4GALNT1. For GM1 rescue, KO‐B4GALNT1 cells received GM1 (50 µM; Sigma‐Aldrich, USA) 2 h before the experimental treatment, with fresh GM1 added every 24 h until the endpoint.

For cross‐model validation, HepG2 and MHCC97H cells were examined together with sorafenib‐resistant derivatives generated using the same intermittent, stepwise dose‐escalation strategy applied to Huh7‐SR. B4GALNT1 perturbation and the downstream drug‐response and cell‐death‐associated assays followed the experimental framework used for the Huh7/Huh7‐SR system.

### 2.5 Construction of stable B4GALNT1 genetic models

Stable overexpression and knockout models were established in the indicated Huh7 or Huh7‐SR background. Full‐length human B4GALNT1 (NCBI RefSeq NM_001478.4) was inserted into pLVX‐Puro, verified by sequencing, and packaged into lentivirus. Cells receiving the corresponding empty vector were processed in parallel as controls. Forty‐eight hours after transduction, puromycin selection (2 µg/mL) was applied for 7 days before expansion and confirmation of B4GALNT1 expression.

For CRISPR/Cas9 knockout, sgRNAs directed against B4GALNT1 exons were inserted into lentiCRISPR v2 and sequence‐confirmed before viral production. A second sgRNA targeting a different B4GALNT1 exon was used to derive independent KO clones in both Huh7 and Huh7‐SR cells. Parental cells were labelled WT, non‐targeting CRISPR controls sgNC, and clones generated with the second B4GALNT1 sgRNA KO‐2. At 48 h after infection, puromycin (2 µg/mL) was applied for 7 days, after which single cells were cloned and expanded.

### 2.6 Establishment of the sorafenib‐resistant Huh7‐SR cell line

Sorafenib resistance was induced by alternating drug exposure with recovery periods while progressively increasing the concentration. Huh7 cultures at approximately 60% confluence were first exposed to 1 µM sorafenib (S7397, Selleck, USA) for 72 h, followed by 4–5 days in drug‐free complete medium. Once growth stabilised, the concentration was raised sequentially to 2, 4, 6, 8, and 10 µM over approximately 6 months. A stable resistant line was considered established when cells could be passaged for more than 10 generations in 10 µM sorafenib without obvious detachment or apoptosis; this derivative was termed Huh7‐SR.

### 2.7 Sorafenib treatment conditions

A 20 mM sorafenib stock was prepared in anhydrous DMSO (Sigma‐Aldrich, D2650, USA), protected from light, and stored at −20°C. Immediately before use, the stock was diluted in serum‐free DMEM to the required concentration. For standard exposure experiments, 5 × 10^3^ cells were plated per well in 96‐well plates, allowed to attach for 12 h, and then cultured with the indicated sorafenib concentration at 37°C/5% CO_2_ for 48 h.

### 2.8 Cell viability assessment using the cell counting Kit‐8 (CCK‐8) assay and IC_50_ calculation

CCK‐8 reagent (Dojindo, CK04, Japan) was used to quantify viable‐cell signal. Ten microliters of reagent was dispensed into each well and incubated for 2 h at 37°C before absorbance was read at 450 nm on a BioTek Synergy HTX reader. Relative viability was calculated as: $\mathrm{Cell}\;\mathrm{viability}\;(\%)=\frac{A_{450\;\mathrm{treated}}}{A_{450\;\mathrm{control}}}\;\times \;100$. Sorafenib IC_50_ values were obtained in GraphPad Prism by fitting a variable‐slope log(inhibitor)‐versus‐response curve.

### 2.9 Calculation of the resistance index (RI)

An RI > 1 indicates reduced sorafenib sensitivity. For genetic‐intervention comparisons, relative resistance was derived by normalising the IC_50_ of each intervention group to its matched control; values above the reference indicate lower drug responsiveness. The resistance index was calculated as: $\mathrm{RI}=\frac{IC_{50(\mathrm{Huh}7-\mathrm{SR})}}{IC_{50\;(\mathrm{Huh}7)}}$

### 2.10 Western blot

Cells or tissue samples were lysed using RIPA lysis buffer supplemented with protease inhibitors (#P0013B, Beyotime, China) for 30 min, and then centrifugation at 12 000 × *g* for 10 min at 4°C to collect the supernatants. Protein concentrations were determined using the bicinchoninic acid (BCA) assay (#P0010, Beyotime, China). Equal amounts of protein (20 µg) were separated by 10% sodium dodecyl sulphate‐polyacrylamide gel electrophoresis (SDS‐PAGE) and transferred onto polyvinylidene fluoride (PVDF) membranes (#IPVH00010, Millipore, USA). Membranes were blocked with 5% non‐fat milk (#232100, BD Biosciences, USA) at room temperature for 1 h and then incubated with primary antibodies at 4°C overnight. The primary antibodies included anti‐B4GALNT1 (ab251713, 1:1000, Abcam, UK), anti‐GPX4 (ab125066, 1:1000, Abcam, UK), anti‐cleaved caspase‐1 (Asp296) (ab179515, 1:1000, Abcam, UK), anti‐GSDMD‐N (N‐terminal) (ab215203, 1:1000, Abcam, UK), anti‐SLC7A11 (PA1‐16893, 1:1000, Thermo Fisher Scientific), and anti‐GAPDH (ab8245, 1:5000, Abcam, UK) as an internal control. The next day, the membranes were exposed to a horseradish peroxidase (HRP)‐conjugated secondary antibody (goat anti‐rabbit IgG–HRP, ab6721, 1:5000, Abcam) at room temperature for 1 h. Protein bands were displayed using an enhanced chemiluminescence (ECL) substrate (ECL Western Blotting Substrate, Thermo Fisher Scientific, USA), imaged with a ChemiDoc MP system (Bio‐Rad, USA), and subsequently quantified using ImageJ software.

### 2.11 Reverse transcription quantitative PCR (RT‐qPCR)

Total RNA was isolated from cultured cells and reverse‐transcribed to complementary DNA (cDNA). Quantitative PCR was carried out with a SYBR Green‐based real‐time PCR workflow with gene‐specific primers for B4GALNT1, GPX4, and SLC7A11. GAPDH served as the internal reference gene. Relative transcript abundance was derived using the 2^−ΔΔCt^ method, with the indicated control group used as the calibrator. Each experiment included three independent biological replicates and technical amplification replicates were averaged before biological‐level statistical analysis.

### 2.12 Fe^2^^+^ fluorescence detection

Intracellular labile Fe^2^^+^ levels were evaluated with the FerroOrange probe (F374, Dojindo, Japan). Following treatment, the cells were rinsed twice with Hanks’ Balanced Salt Solution (HBSS; Gibco) and incubated with 1 µM FerroOrange working solution at 37°C for 30 min. Cells were subsequently washed twice with phosphate‐buffered saline (PBS) and immediately examined using a fluorescence microscope (Zeiss LSM 900). Fluorescence settings were configured as 543 nm for excitation and 580 nm for emission. At least five fields of view or 10 000 cells per group were acquired for quantitative analysis.

### 2.13 Lipid peroxidation staining (C11‐BODIPY™ 581/591)

For evaluation of lipid peroxidation in cells, the lipid peroxidation fluorescent probe C11‐BODIPY™ 581/591 (D3861, Thermo Fisher Scientific, USA) was applied. For cellular samples, cells were washed once with PBS after treatment, incubated with C11‐BODIPY working solution at a final concentration of 5 µM at 37°C for 30 min in the dark. At the end of incubation, cells were rinsed twice with PBS before immediate fluorescence imaging. For paraffin‐embedded tissue sections, after deparaffinisation with xylene, rehydration through graded ethanol, and washing with PBS, 5 µM C11‐BODIPY solution was added and incubated at 37°C for 30 min in the dark, and subsequently washed three times with PBS. Fluorescence was examined using a Zeiss LSM 900 confocal microscope (Zeiss, Germany) with 488 nm excitation, and both green fluorescence (oxidised form; Em ∼510 nm) and red fluorescence (non‐oxidised form; Em ∼590 nm) were acquired. Lipid peroxidation was quantified as the ratio of green/(green + red) fluorescence.

### 2.14 Lactate dehydrogenase (LDH) assay

For quantification of extracellular LDH release, 200 µL of culture supernatant was harvested and centrifuged at 400 × *g* for 5 min. Thereafter, 120 µL of the post‐centrifugation supernatant was transferred into a 96‐well plate. Supernatant from Huh7 cells treated with Triton X100 (Sigma‐Aldrich, Shanghai, China) served as the positive control wells. Thereafter, 60 µL of LDH working reagent (BA1800, Shangbao Biotech, Shanghai, China) was introduced into each well, mixed gently, and incubated at room temperature for 30 min in the dark (wrapped in aluminium foil and placed on a horizontal shaker at low speed). Absorbance was measured at 490 nm, with 600 nm used as the reference wavelength for dual‐wavelength reading.

### 2.15 Malondialdehyde (MDA) measurement

For determination of MDA levels as an index of lipid peroxidation, an MDA assay kit (S0131, Beyotime, China) was used in accordance with the supplier's protocol. Following treatment, cells were lysed in the kit lysis buffer, centrifuged at 10 000 × *g* for 10 min to collect the supernatant. Quantified aliquots were subsequently incubated with the provided reagents for 40 min in a 95°C water bath. After cooling to room temperature, absorbance at 532 nm was measured. MDA content was quantified with a standard curve and reported as µmol MDA/mg total protein.

Total cellular ROS and mitochondrial superoxide were assessed in the second‐sgRNA validation experiments using DCF‐DA and MitoSOX fluorescence, respectively. Cells were stained according to the manufacturers’ instructions, washed to remove unbound probe, and imaged under identical acquisition settings across matched groups. Propidium iodide (PI) staining served as a membrane‐integrity/cell‐death readout. Fluorescence intensities or PI‐positive fractions were quantified from matched fields using identical analysis thresholds across WT, sgNC, and KO‐2 groups. These assays were conducted in Huh7 and Huh7‐SR backgrounds in three independent biological experiments.

### 2.16 Measurement of glutathione (GSH) and GSH/oxidised glutathione (GSSG) ratio

Intracellular GSH, GSSG, and their ratio were quantified with a total GSH assay kit (S0053, Beyotime, China). Following treatment, the cells were harvested and fully lysed with the protein removal reagent provided in the kit, and then centrifugation at 12 000 × *g* for 10 min at 4°C; the supernatant was subsequently used for subsequent measurements.

For total GSH (GSH + GSSG) measurement, an aliquot of the supernatant was mixed with the working solution, loaded into a 96‐well plate, and incubated at room temperature in the dark for 30 min. The absorbance was subsequently measured at 412 nm using a microplate reader, and the total GSH content was derived based on the standard curve.

For GSSG‐specific measurement, another aliquot of the supernatant was derivatised with a GSH scavenger (2‐vinylpyridine) to block free GSH and thereby retain only GSSG for the reaction. Thereafter, the absorbance was measured, and the GSSG content was derived as described above.

The reduced GSH content was derived using the following equation: GSH = Total GSH − 2 × GSSG. Final values were reported as nmol GSH/mg protein and nmol GSSG/mg protein, and the GSH/GSSG ratio was derived to indicate the intracellular redox status. The total protein concentration was quantified with the BCA method (Beyotime, China) for normalisation.

### 2.17 Adenosine triphosphate (ATP) content measurement

For evaluation of cellular energy metabolism across different treatment groups, we measured intracellular ATP using an ATP Assay Kit (ab83355, Abcam, UK). Following treatment, cells were harvested from 6‐well plates, lysed in the kit‐provided lysis buffer for 10 min, and centrifuged at 12 000 × *g* and 4°C for 10 min to collect the supernatant. A defined volume of sample (e.g., 50 µL) was mixed with the working reagent and incubated at room temperature for 5 min; fluorescence was subsequently recorded on a microplate reader (Synergy H1, BioTek, USA) at ∼535 nm excitation and ∼587 nm emission. An ATP standard curve was generated to calculate ATP levels in each group, expressed as nmol ATP per mg total protein.

### 2.18 Mitochondrial ultrastructure observation

For characterisation of mitochondrial morphology and to assess the extent of injury and repair, cells were fixed in 2.5% glutaraldehyde (G5882, Sigma‐Aldrich, USA) at 4°C for 2 h. The fixed cells were subsequently washed three times with .1 M PBS (pH 7.4) for 5 min each. The next step was to samples were post‐fixed with 1% osmium tetroxide for 1 h, dehydrated through a graded ethanol series (30%, 50%, 70%, 90%, and 100%; 10 min each), cleared with ethyl acetate for 10 min, and embedded in epoxy resin. Ultrathin sections were sequentially stained with 1% uranyl acetate and 1% lead citrate for 30 min per step at room temperature in the dark. Finally, mitochondrial ultrastructure was examined by transmission electron microscopy (TEM; JEM‐2100F, JEOL, Japan) at an accelerating voltage of 200 kV, and 5–10 randomly selected fields per group were imaged for morphological documentation.

### 2.19 Mitochondrial membrane potential (JC‐1) measurement

Mitochondrial membrane potential was evaluated with a JC‐1 mitochondrial membrane potential assay kit (T3168, Thermo Fisher Scientific, USA). After the designated treatments, cells were incubated in 6‐well plates with JC‐1 dye (2 µM) at 37°C for 20 min. After two washes with PBS, red (J‐aggregate, ∼590 nm) and green (J‐monomer, ∼530 nm) fluorescence signals were acquired using a fluorescence microscope (Zeiss LSM 900, Germany). The red/green fluorescence ratio was derived for each group as an index of mitochondrial membrane potential.

### 2.20 Enzyme‐linked immunosorbent assay (ELISA) measurement

ELISA was conducted to quantify IL‐1β and IL‐18 in culture supernatants collected after 24 h of cell culture. More specifically, 100 µL of each sample was introduced into each well and analysed using the following ELISA kits: IL‐1β ELISA kit (ab185986, Abcam, UK) and IL‐18 ELISA kit (ab215539, Abcam, UK). All procedures were conducted in accordance with the supplier's protocol. After incubation at room temperature for 2 h, the liquid was discarded, and the wells were washed six times with washing buffer (300 µL/well), with residual liquid removed by gently tapping on absorbent paper after each wash. Thereafter, 100 µL of HRP‐labelled streptavidin working solution was added and incubated at room temperature for 45 min; the wells were subsequently washed again. Next, 100 µL of substrate solution was added and incubated for 15 min at room temperature in the dark. Finally, 100 µL of stop solution was introduced into each well, and absorbance was read at 450 nm within 30 min using a microplate reader (Multiskan FC, Thermo Fisher Scientific, USA).

### 2.21 Immunofluorescence analysis

Immunofluorescence was employed to assess protein abundance, subcellular localisation, and colocalisation. Cells were seeded on sterile coverslips, subjected to the indicated treatments, fixed with 4% paraformaldehyde for 10 min, washed with PBS, permeabilised with .1% Triton X‐100 when intracellular antigen access was required, and blocked with 1% bovine serum albumin for 1 h. Primary antibodies against B4GALNT1, GPX4, cleaved caspase‐1, or GSDMD‐N were incubated overnight at 4°C, and then the appropriate Alexa Fluor‐conjugated secondary antibodies. Nuclei were counterstained with DAPI. GM1 was displayed with fluorescent cholera toxin B subunit (CTxB) under non‐permeabilising conditions when membrane‐associated GM1 was assessed. For CASP1–GM1 colocalisation, dual‐channel images were acquired under identical settings and Pearson's correlation coefficient was derived in ImageJ. GSDMD‐N membrane localisation was quantified from matched fields using a uniform analysis threshold. Three to five randomly selected fields were analysed per biological replicate.

### 2.22 Flow‐cytometric annexin V/propidium iodide (PI) assay

Apoptosis was quantified and morphologically visualised using a dual‐staining strategy with Annexin V‐fluorescein isothiocyanate (FITC) and PI. More specifically, cells were stained with the Annexin V‐FITC/PI Apoptosis Detection Kit (BD Biosciences, USA). Following treatment, both adherent cells and floating cells in the culture supernatant were harvested together, washed twice with ice‐cold PBS, and centrifuged at 300 × *g* for 5 min. The cell pellet was resuspended in 1× Binding Buffer and adjusted to a density of 1 × 10^6^ cells/mL. Thereafter, 100 µL of cell suspension was exposed to 5 µL Annexin V‐FITC and 5 µL PI for 15 min at room temperature in the dark with gentle mixing. Then, 400 µL of 1× Binding Buffer was introduced into terminate the reaction, and samples were immediately analysed. Data acquisition was conducted on a flow cytometer (BD FACSCanto II, BD Biosciences, USA) using 488 nm excitation; FITC fluorescence was harvested in FL1 (530/30 nm) and PI fluorescence was harvested in FL3 (> 600 nm). At least 10 000 events were acquired per sample. Measurements were analysed with FlowJo software (v10.8.1) to determine the proportions of apoptotic and viable cells. Additionally, for morphological visualisation, cells were seeded onto coverslips in 24‐well plates and stained with Annexin V‐FITC/PI as described above, and then imaging using a fluorescence microscope (Olympus IX73, Japan). FITC signals (green) indicated early apoptotic cells, whereas PI signals (red) indicated late apoptotic and necrotic cells. Five random fields were imaged for each group to illustrate apoptosis‐associated morphological differences among treatments.

### 2.23 EdU incorporation assay

DNA synthesis was assessed by 5‐ethynyl‐2′‐deoxyuridine (EdU) incorporation. Cells were exposed to the indicated sorafenib conditions and incubated with EdU labelling solution according to the assay‐kit instructions. After labelling, cells were fixed, permeabilised, and subjected to fluorescent click‐reaction detection; nuclei were counterstained with DAPI. Images were acquired under identical exposure settings across matched groups. EdU‐positive nuclei were quantified as a percentage of total DAPI‐positive nuclei from matched fields, and three independent biological experiments were analysed.

### 2.24 Colony‐formation assay

In brief, cells in the logarithmic growth phase were dissociated with .25% Trypsin–ethylenediaminetetraacetic acid (EDTA) (Gibco, 25200056, USA) to generate a single‐cell suspension, and viability was evaluated by Trypan Blue staining (Thermo Fisher Scientific, USA) to ensure a viability of > 95%. Thereafter, cells were seeded into 6‐well plates at 500–1000 cells/well in 2 mL complete medium (DMEM, Gibco 11965092, USA) supplemented with 10% FBS (Gibco 10099141, USA) and 1% Penicillin‐Streptomycin (Gibco 15140122, USA). Cells were incubated for 14 days at 37°C with 5% CO_2_ in a humidified incubator (Thermo Forma Series II, USA), and the medium was replaced every 3 days. When individual colonies reached approximately 50–100 µm in diameter, the medium was carefully removed; cells were rinsed twice with PBS and fixed with 4% PFA (Sigma‐Aldrich, USA) for 15 min. After one PBS wash, cells were stained with .1% crystal violet solution (Solarbio, G1063, China) for 20 min at room temperature. After staining, the plates were gently rinsed with tap water until the background was clear, air‐dried completely, and subjected to data acquisition. Colony numbers were acquired by photographing the plates and automatically quantified using ImageJ software. Under unified threshold conditions, cell clusters with an area >.01 mm^2^ were defined by valid colonies. Each experiment was conducted in triplicate. The final evaluation metrics included total colony number and colony formation rate (number of colonies/number of seeded cells × 100%).

### 2.25 Lectin staining

For detection of the expression of specific cell surface glycan structures (Tn antigen, O‐glycan, N‐glycan, and terminal Gal residues), lectin fluorescence staining analysis was conducted on the cells. Fluorescently labelled lectins were used in the experiment, including FITC‐vicia villosa agglutinin (VVA; Vector Laboratories, FL‐1231, USA), FITC‐soybean agglutinin (SBA) (Vector Laboratories, FL‐1011, USA), Alexa Fluor 488‐labelled wheat germ agglutinin (WGA; Thermo Fisher Scientific, W11261, USA), and FITC‐Ricinus Communis Agglutinin I (RCA‐I) (Vector Laboratories, FL‐1081, USA). All operations were conducted in the dark. Cells were cultured in 12‐well plates or on coverslips to approximately 70%–80% confluence, after which the medium was discarded, and cells were rinsed twice with ice‐cold PBS (Gibco, 10010023, USA). Thereafter, cells were fixed with 4% PFA (Sigma‐Aldrich, P6148, USA) for 15 min. After fixation, cells were washed once with PBS. For detection of intracellular glycan structures, cells were permeabilised with .1% Triton X‐100 (Solarbio, T8200, China) for 5 min at room temperature; if only cell membrane surface glycans were detected, the permeabilisation step was omitted. The next step was to cells were blocked with 5% BSA (Solarbio, A8020, China) for 1 h at room temperature to reduce nonspecific binding. Each fluorescently labelled lectin was diluted to a final concentration of 5–10 µg/mL in accordance with the supplier's protocol, added to the cells, and incubated for 30 min at room temperature. At the end of incubation, cells were rinsed three times with PBS for 5 min each to remove unbound lectins. Thereafter, anti‐fluorescence quenching mounting medium Fluoromount‐Gold (Invitrogen, 0100–01, USA) was added dropwise for mounting. Fluorescence signals were acquired using a confocal microscope (Leica TCS SP8, Germany) with a unified excitation at 488 nm and emission at 500–550 nm for imaging. All images were acquired under identical exposure, gain, and light intensity parameters. Quantitative analysis was carried out with ImageJ software to determine the mean grey value of the cells.

### 2.26 Lectin blot

For detection of changes in the overall cellular protein glycosylation phenotype, lectin blot analysis was conducted. Cells after different treatments were placed on ice and lysed for 30 min in RIPA lysis buffer (Beyotime, P0013B, China) containing 1× Protease Inhibitor Cocktail (Roche, 04693159001, Switzerland), and then centrifugation at 12 000 × *g* for 15 min to collect the supernatant. Protein concentration was determined using a BCA protein assay kit (Thermo Fisher Scientific, 23225, USA). For each sample, 50 µg of total protein was taken, mixed with 5× SDS‐PAGE loading buffer (without reducing agent), and loaded after denaturation at 65°C for 5 min. Proteins were separated by 10% SDS‐PAGE and transferred to a PVDF membrane (Millipore, IPVH00010, USA) at 300 mA for 1.5 h. After transfer, the PVDF membrane was blocked with 5% non‐fat milk (prepared in tris‐buffered saline with tween 20 [TBST]) for 1 h at room temperature to reduce nonspecific binding of lectins. After blocking, Biotin‐labelled lectins, including Biotin‐VVA (Vector Laboratories, B‐1231, USA), Biotin‐SBA (Vector Laboratories, B‐1015, USA), and Biotin‐PNA (Vector Laboratories, B‐1075, USA), were added at a working concentration of 1–3 µg/mL and incubated for 1.5 h at room temperature. Thereafter, the membrane was rinsed three times with TBST for 10 min each.

Thereafter, HRP‐labelled Streptavidin (secondary antibody, Vector Laboratories, SA‐5004, USA, 1:5000) was added and incubated for 1 h at room temperature. Following washing the membrane with TBST, ECL substrate (Thermo Fisher Scientific, 32106, USA) was added, and exposure detection was conducted on a ChemiDoc MP imaging system (Bio‐Rad, USA). To verify the consistency of protein loading in each lane, Ponceau S staining was first performed after transfer to record total protein bands as a loading control. Lectin band signals were subjected to densitometric analysis using ImageJ software and normalised to the Ponceau S total protein signal.

### 2.27 O‐glycan structure analysis

To systematically characterise alterations in O‐glycosylation structures across distinct treatment conditions, O‐glycans were released via chemical β‐elimination and subsequently profiled qualitatively and quantitatively using hydrophilic interaction liquid chromatography (HILIC) enrichment coupled with nano‐liquid chromatography‐tandem mass spectrometry (LC‐MS/MS). After cells were washed with PBS (Gibco, 10010023, USA), they were lysed on ice for 30 min in RIPA lysis buffer (Beyotime, P0013B, China) supplemented with 1× protease inhibitor cocktail (Roche, 04693159001, Switzerland). Protein concentrations were subsequently determined using a BCA protein assay kit (Thermo Fisher Scientific, 23225, USA), and 100 µg of total protein per group was subjected to O‐glycan release. More specifically, protein samples were exposed to a mixture containing .1 M NaOH and 1 M NaBH_4_ (Sigma‐Aldrich, USA) at 45°C for 16 h to perform β‐elimination, with continuous shaking to ensure reaction homogeneity. The reaction was subsequently quenched by adding glacial acetic acid (Sigma‐Aldrich, USA), and then desalting and peptide removal using C18 solid‐phase extraction cartridges (Waters, Sep‐Pak C18, USA). Samples were concentrated to 50 µL under nitrogen (Thermo Fisher Scientific, USA). To enrich O‐glycans, HILIC solid‐phase extraction cartridges (Waters, Oasis HILIC, USA) were used. The columns were conditioned and loaded with samples, washed with 80% acetonitrile/.1% trifluoroacetic acid (TFA), and eluted with .1% TFA in water. Finally, eluates were vacuum‐dried and reconstituted in 10 µL of .1% formic acid in downstream analysis.

For determination of O‐glycan structural features, nano‐LC‐MS/MS was carried out with a dedicated analytical platform. Separation was conducted on an EASY‐nLC 1200 system (Thermo Fisher Scientific, USA) equipped with an Acclaim PepMap RSLC C18 column (75 µm × 25 cm, 2 µm) at a flow rate of 300 nL/min. The gradient increased linearly from 2% B (acetonitrile containing .1% formic acid) to 35% B over 45 min, and then an increase to 80% B over 5 min and a 5‐min hold to wash the column. Mass spectrometry was carried out on an Orbitrap Fusion Lumos instrument (Thermo Fisher Scientific, USA), with full MS acquired in the Orbitrap at a resolution of 120 000 over an *m*/*z* range of 400–1800. MS/MS spectra were acquired using higher‐energy collisional dissociation (HCD) with a collision energy of 28%, an MS2 resolution of 30 000, an automatic gain control (AGC) target of 2 × 10^5^, and a maximum injection time of 80 ms. Raw data were processed using Byonic (v4.2, Protein Metrics Inc., USA) and GlycoWorkbench (v2.1) for glycan identification. The search space included core 1 and core 2 O‐glycan libraries, with oxidation/dehydration events and diagnostic fragmentation and neutral‐loss features for GalNAc, Gal, and NeuAc enabled. Quantification was conducted by peak‐area integration, and all O‐glycan signals were normalised against the total glycan peak area.

### 2.28 Ganglioside profiling by thin‐layer chromatography and targeted LC‐MS/MS

For thin‐layer chromatographic profiling, the ganglioside‐containing lipid fraction was dried under nitrogen, reconstituted in chloroform/methanol, and applied to silica gel 60 high‐performance TLC plates together with GM3, GM2, and GM1 standards. Plates were developed with chloroform/methanol/.2% aqueous CaCl_2_ (55:45:10, v/v/v), air‐dried, and visualised with resorcinol reagent. Band intensities were quantified by densitometry using identical background correction across lanes. Targeted LC‐MS/MS served as the complementary quantitative method described below.

To quantitatively profile ganglioside abundance (GM1, GM2, and GM3) under different treatment conditions, samples were analysed with organic solvent extraction and then targeted LC‐MS/MS. Following treatment, cells were rinsed twice with ice‐cold PBS (Gibco, 10010023, USA) and harvested as pellets, using approximately 1 × 10^6^ cells per analytical unit. Cell pellets were lysed in methanol containing an internal standard, namely C17‐GM1 (Avanti Polar Lipids, 860067, USA), at a final amount of 50 pmol per sample. Thereafter, chloroform (LC‐MS grade, Thermo Fisher Scientific, USA) and water were introduced into generate a biphasic system at a methanol:chloroform:water ratio of 2:2:1.5 (v/v/v), and the mixture was vortexed for 1 min and centrifuged at 12 000 × *g* for 10 min to collect the lower organic phase. To improve recovery, chloroform extraction was repeated once, and the two organic phases were combined and evaporated to dryness at room temperature under nitrogen (Thermo Fisher Scientific, USA). The residue was subsequently reconstituted in 100 µL of methanol/water (70:30, v/v) containing .1% formic acid, filtered through a .22 µm organic membrane, and subjected to LC‐MS/MS analysis.

To achieve robust chromatographic separation for targeted ganglioside quantification, analysis was carried out with a Vanquish UHPLC system (Thermo Fisher Scientific, USA) coupled to an ACQUITY UPLC BEH C18 column (2.1 × 100 mm, 1.7 µm; Waters, USA). Mobile phase A consisted of water/methanol (50:50, v/v) containing .1% formic acid and 5 mM ammonium formate, whereas mobile phase B comprised isopropanol/methanol (90:10, v/v) containing .1% formic acid and 5 mM ammonium formate; the flow rate was set to .25 mL/min. The gradient was programmed as follows: 0–2 min, 40% B; 2–18 min, linear increase from 40% to 90% B; 18–23 min, hold at 90% B; and 23–25 min, return to 40% B and then column re‐equilibration. The injection volume was 5 µL, and the column temperature was maintained at 40°C.

Targeted mass spectrometry was conducted on a triple quadrupole mass spectrometer, TSQ Altis (Thermo Fisher Scientific, USA), with data acquired in electrospray ionisation negative mode (ESI−). The ion‐source parameters were set to a spray voltage of 2.7 kV and a capillary temperature of 300°C, whereas the sheath‐ and auxiliary‐gas flow rates were configured according to the manufacturer's recommended settings. Targeted acquisition was conducted in multiple reaction monitoring (MRM) mode, with ganglioside‐specific precursor/product ion transitions defined for each analyte. The transitions were *m*/*z* 1546.9→290.1 for GM1, 1384.8→290.1 for GM2, and 1179.7→290.1 for GM3. Collision energies were set to 25–35 eV and were further optimised using authentic standards. Data acquisition and peak‐area integration were completed using Xcalibur and TraceFinder software (Thermo Fisher Scientific, USA).

Quantitative analysis was conducted by first constructing calibration curves using a series of ganglioside standards at known concentrations (Avanti Polar Lipids, USA) together with a fixed concentration of the C17‐GM1 internal standard. To ensure linearity and reproducibility, each calibration point was injected at least in duplicate. Thereafter, the molar concentration of each ganglioside in samples was derived by inputting the peak‐area ratio (analyte/internal standard) into the corresponding standard curve, and the results were normalised against cell number and reported as pmol/10^6^ cells.

### 2.29 Enrichment analysis of glycosphingolipids in lipid‐raft microdomains

For quantification of the enrichment of glycosphingolipids (GM1, GM2, and GM3) in plasma membrane lipid rafts, detergent‐resistant membranes (DRMs) were isolated using cold Triton X‐100 extraction, and then sucrose density‐gradient ultracentrifugation and LC‐MS/MS‐based quantification. All procedures were conducted at 4°C to preserve lipid‐raft integrity. Cells at approximately 80% confluence were rinsed twice with ice‐cold PBS (Gibco, 10010023, USA) and harvested by scraping. The cell pellet was resuspended in 1 mL lipid‐raft lysis buffer (25 mM Tris‐HCl, pH 7.5; 150 mM NaCl; 5 mM EDTA) supplemented with 1% Triton X‐100 (Sigma‐Aldrich, t9284, USA) and 1× protease inhibitor cocktail (Roche, 04693159001, Switzerland). The lysate was gently incubated on a rocker at 4°C for 30 min and then repeatedly passed through a 25 g needle 10 times to facilitate lysis. Next, the lysate was mixed with an equal volume of 80% sucrose (w/v, prepared in the buffer) and loaded at the bottom of a 12 mL ultracentrifuge tube, and then sequential overlay with 30% and 5% sucrose to form a discontinuous gradient. The gradients were ultracentrifuged in an ultracentrifuge (Beckman Coulter optima l‐90k, USA) using an SW41Ti rotor at 200 000 × *g* for 18 h. Following centrifugation, 12 fractions of 1 mL were harvested from top to bottom; fractions 3–5 were defined by the lipid‐raft–enriched DRMs region, whereas fractions 9–12 were designated as the non‐raft soluble region.

For validation of the efficiency of lipid‐raft isolation, equal volumes from each fraction were analysed for caveolin‐1 (Cell Signaling Technology, 3267s, USA) or flotillin‐1 (Cell Signaling Technology, 18634s, USA) as lipid‐raft markers. After the lipid‐raft region was identified, the corresponding gradient fractions were harvested for glycosphingolipid measurements.

For quantification of ganglioside abundance, lipids were extracted with chloroform/methanol (2:1, v/v) and then analysed on a TSQ Altis (Thermo Fisher Scientific, USA) triple quadrupole mass spectrometer operating in negative‐ion ESI mode with MRM scanning. More specifically, the MRM transitions for GM1, GM2, and GM3 were set to 1546.9→290.1, 1384.8→290.1, and 1179.7→290.1, respectively. Therefore, C17‐GM1 (avanti polar lipids, 860067, USA) served as an internal standard for quantitative normalisation across all samples. Additionally, glycosphingolipid levels in lipid‐raft and non‐lipid‐raft fractions were derived using the peak area ratio (analyte/is) and were normalised against the total protein content of each fraction as measured by the BCA assay (Thermo Fisher Scientific, 23225, USA).

For orthogonal lipid‐raft perturbation, WT, KO‐B4GALNT1, Oe‐NC, and Oe‐B4GALNT1 cells were treated with methyl‐β‐cyclodextrin (MβCD; 10 mM, 30 min). In the cholesterol‐repletion condition, cholesterol (.5 mM) was supplied after MβCD treatment before detergent‐resistant membrane analysis. GM1 enrichment in DRM fractions was quantified, and Flotillin‐1 and Caveolin‐1 served as raft‐associated protein markers together with cholera toxin B (CTxB)‐based detection of GM1. In a separate ferroptosis pharmacology module, cells were exposed to erastin (5 µM, 24 h), with or without ferrostatin‐1 (1 µM), and PI‐positive cells were quantified as a functional cell‐death readout. Raft‐perturbation experiments and the erastin/ferrostatin‐1 module were conducted as separate validation experiments.

### 2.30 Ethics approval and housing conditions for animal experiments

All animal procedures were performed under the institutional animal protocol approved by the Animal Ethics Committee of The Seventh People's Hospital Affiliated to Shanghai University of Traditional Chinese Medicine (No. 2025‐AR‐033) and followed the Guide for the Care and Use of Laboratory Animals, 8th edition. Animal numbers were kept to the minimum required for the study, and measures to limit procedure‐related pain and distress were incorporated throughout.

Female SPF BALB/c nude mice (4–6 weeks old; strain 211; 20–22 g) were obtained from Beijing Vital River Laboratory Animal Technology Co., Ltd. Animals were maintained in a barrier facility at 22 ± 2°C and 50%–60% relative humidity on a 12‐h light/dark cycle, with free access to sterilised chow and ultrafiltered sterile water. Cages, bedding, work surfaces, and personnel procedures were managed under routine barrier‐housing practices.

Mice were allowed to acclimate for at least 7 days before any intervention. Trained personnel conducted all procedures under the approved protocol, using anaesthesia or analgesia when required. General condition, body weight, and signs of procedure‐related distress were monitored throughout the experiment.

### 2.31 Establishment and grouping of the subcutaneous tumourigenesis model

Female BALB/c nude mice aged 4–6 weeks were acclimated for 1 week and then randomly assigned to groups. All cells used in this experiment were sorafenib‐resistant HCC cells (Huh7‐SR) engineered into stable B4GALNT1 intervention cell lines, including Vector control–resistant cells, Oe‐B4GALNT1‐resistant cells, KO‐B4GALNT1‐resistant cells, and KO‐B4GALNT1+GM1 rescue–resistant cells. Vector control cells, Oe‐B4GALNT1 cells, KO‐B4GALNT1 cells, and KO‐B4GALNT1+GM1 rescue cells in logarithmic growth were dissociated and collected, resuspended in PBS, and mixed with Matrigel (1:1, v/v). Then, 1 × 10^6^ cells in a total volume of 100 µL were slowly injected subcutaneously into the right axilla of each mouse to generate subcutaneous xenograft models. For the GM1 rescue group, GM1 (20 mg/kg, Sigma‐Aldrich, USA) was administered by intraperitoneal injection 45 min before cell inoculation and was replenished every 3 days thereafter. After modelling, nude mice were returned to their cages for continued housing, and their general condition and the injection site were inspected daily. When tumour volume reached 100 mm^3^, nude mice in each group received daily intraperitoneal injections of sorafenib (30 mg/kg, Selleck Chemicals, USA) for 10 consecutive days. During treatment, tumour volume and body weight of the nude mice were monitored as described above.

### 2.32 Monitoring of tumour volume and terminal sampling

Tumour length (L) and width (W) were measured every 7 days, and tumour volume was derived using the formula (*L* × *W* ^2^/2). Monitoring continued until day 28; thereafter, nude mice were euthanised by intraperitoneal injection of an overdose of sodium pentobarbital (100 mg/kg), and tumours were excised, weighed, and sectioned for subsequent histology and immunohistochemistry.

### 2.33 Pathological experiments

Subcutaneous xenograft tumour tissues were fixed in 4% PFA, subjected to gradient dehydration, and embedded in paraffin. Thereafter, 3‐µm serial sections were cut and baked for subsequent use.

Haematoxylin and eosin (H&E) staining: Paraffin sections were deparaffinised and rehydrated. Nuclei were stained with haematoxylin (H8070, Solarbio, Beijing, China), and the cytoplasm was stained with eosin (G1100, Solarbio, Beijing, China). Sections were subsequently dehydrated and mounted, and images were acquired under a microscope for data analysis.

Immunohistochemistry: Paraffin sections were deparaffinised and rehydrated. Tissue sections were placed in an antigen retrieval box containing EDTA antigen retrieval buffer (pH 9.0) (C9999, Merck, USA) and heated in a microwave for antigen retrieval. After cooling, the slides were placed in PBS (pH 7.4) and washed three times on a decolourising shaker for 15 min each. Endogenous peroxidase activity was blocked with 3% hydrogen peroxide solution (H1009, Merck, USA), and non‐specific binding was blocked with serum BSA. Properly diluted primary antibodies against cleaved caspase‐1 (1:100, ab179515, Abcam, UK) and GSDMD‐N (1:100, ab215203, Abcam, UK) were introduced into the sections, which were subsequently placed flat in a humidified chamber and incubated overnight at 4°C. After shaking off the excess liquid, the corresponding HRP‐conjugated goat anti‐rabbit secondary antibody (1:200, GB23303, Servicebio, Wuhan, China) from the immunohistochemistry kit was added dropwise within the circle to cover the tissue, and then incubation at room temperature for 30–50 min. Thereafter, freshly prepared 3,3′‐diaminobenzidine (DAB) colour development solution was added. Sections were counterstained with haematoxylin (H8070, Solarbio, Beijing, China) for approximately 3 min, dehydrated, and mounted. Finally, images were captured under a microscope for observation, acquisition, and analysis, and the proportion of intracellular positivity was derived.

### 2.34 Local clinical cohort and B4GALNT1 H‐score analysis

The local clinical series included 92 patients with HCC who were treated with sorafenib during 2022–2023 and had both tumour tissue and follow‐up information available. B4GALNT1 immunostaining was scored on a 0–300 H‐score scale, and the cohort was split at the median into low‐ and high‐expression groups (*n* = 46 each). Response categories were assigned from clinical and radiologic records as partial response, stable disease, poor response, or progressive disease; partial response/stable disease were grouped as disease control, whereas poor response/progressive disease formed the adverse‐response category. Progression‐free survival was measured from sorafenib initiation to progression or death, and overall survival from sorafenib initiation to death or last follow‐up. Kaplan–Meier curves were compared by log‐rank test, and Cox regression was used to estimate hazard ratios and 95% confidence intervals for B4GALNT1 expression together with age, AJCC stage, T stage, vascular invasion, and sorafenib response.

### 2.35 Time‐resolved membrane‐permeability, caspase‐inhibitor, and ferrostatin‐1 rescue assays

Time‐resolved membrane‐permeability, caspase‐inhibitor, and ferrostatin‐1 rescue experiments were conducted in Huh7‐SR cells. For membrane‐permeability measurements, Vector, Oe‐B4GALNT1, KO‐B4GALNT1, and KO‐B4GALNT1 + GM1 cells were exposed to sorafenib (10 µM). PI‐positive and YO‐PRO‐1‐positive cell fractions were quantified at serial time points during the first 240 min, and LDH release was measured over 24 h. Cleaved caspase‐3 fluorescence intensity was quantified at the endpoint as an apoptosis‐associated control. For caspase‐dependence analysis, cells were treated with DMSO, VX‐765 (10 µM), Ac‐YVAD‐cmk (20 µM), Z‐DEVD‐fmk (20 µM), or Z‐VAD‐fmk (20 µM), followed by quantification of YO‐PRO‐1‐positive cells. For ferrostatin‐1 rescue, separate KO and overexpression modules were evaluated under sorafenib treatment. Each module included Vector + Sorafenib, Vector + Sorafenib + ferrostatin‐1 (1 µM), the corresponding KO‐B4GALNT1 or Oe‐B4GALNT1 + Sorafenib group, and that group + ferrostatin‐1 (1 µM). Lipid ROS was quantified at the endpoint, whereas cell viability, ATP, and LDH release were followed for 24 h. YO‐PRO‐1 fluorescence was additionally monitored over 24 h to determine the time to the first detectable YO‐PRO‐1‐positive event. At 24 h, paired YO‐PRO‐1 and PI staining was used to determine the percentage of YO‐PRO‐1‐positive cells that continued to exclude PI. All experiments were performed in three independent biological replicates.

### 2.36 Genetic rescue and catalytic‐dead B4GALNT1 validation

To determine whether rescue required B4GALNT1 catalytic activity, Huh7‐SR‐derived KO‐B4GALNT1 cells were transduced with empty vector, wild‐type B4GALNT1 cDNA, or a B4GALNT1‐D358A construct. Asp358 is located within the conserved D356–D358 DxD catalytic motif. Earlier mutagenesis showed that substitution of D356 or D358 reduces GM2 synthase activity to background levels,^23^ and recent structural and biochemical characterisation demonstrated that D358 contributes to the Mn^2^^+^‐dependent catalytic environment and that D358A is properly folded but retains < 2% of wild‐type activity.^24^ The D358A substitution was introduced by site‐directed mutagenesis, and the complete insert was sequence‐verified before lentiviral packaging. Stable rescue populations were generated using the same transduction and puromycin‐selection workflow as the other B4GALNT1 models. GM1 enrichment was quantified in detergent‐resistant membrane fractions using CTxB‐based detection according to the raft‐fractionation workflow described above. GPX4, SLC7A11, cleaved CASP1, and GSDMD‐N were quantified by Western blot, normalised to the corresponding loading control, and expressed relative to the KO + Empty vector reference. Sorafenib sensitivity was determined by CCK‐8 dose–response analysis with IC50 fitting, and relative IC50 values were expressed against the KO + Empty vector reference. All rescue experiments were conducted in three independent biological replicates.

### 2.37 Statistical analysis

Quantitative data are presented as mean ± SD. Statistical analyses were performed in GraphPad Prism (v9.5; GraphPad Software, USA). Two‐group comparisons were analysed using unpaired two‐tailed Student's *t*‐tests. Comparisons among three or more independent groups were analysed by one‐way ANOVA followed by Tukey's multiple‐comparisons test. Datasets containing both cell‐line and genotype or treatment factors were analysed by two‐way ANOVA followed by Sidak's multiple‐comparisons test. Time‐course datasets were analysed by two‐way ANOVA with Sidak's multiple‐comparisons test, and tumour‐volume growth curves were analysed by repeated‐measures ANOVA. Survival distributions were compared using the log‐rank test. Categorical variables were analysed using the chi‐square test or Fisher's exact test, and non‐normally distributed H‐score values were compared using the Mann–Whitney *U* test. All tests were two‐sided, with *p* < .05 considered statistically significant. Significance levels were defined as *p < .05, **p < .01, ***p < .001, and ****p < .0001. Biological replicate numbers are reported in the corresponding figure legends.

## 3 RESULTS

### 3.1 B4GALNT1 expression in HCC and its association with clinical features and outcome

Within TCGA‐LIHC, B4GALNT1 transcript levels were higher in HCC than in adjacent or other non‐tumour liver samples (Figure S1A). A separate comparison based on tumour status showed the same overall direction (Figure S1B), supporting a tumour‐associated increase in B4GALNT1 expression.

B4GALNT1 abundance also differed across clinicopathological categories. Expression generally rose with advancing pathological T stage and overall stage (Figure S1C and D), and N1 cases showed higher values than N0 cases (Figure S1E). No clear M‐stage trend was inferred because the metastatic subgroup was small; the comparison involving M1 was therefore treated cautiously (Figure S1F).

Patients in the B4GALNT1‐high stratum had shorter overall survival than those in the low‐expression stratum (Figure S1G). Expression was likewise higher among patients who experienced an overall‐survival event than among censored patients (Figure S1H). ROC analysis further showed that B4GALNT1 separated tumour from non‐tumour samples with an AUC of approximately .915 (Figure S1I). These cohort‐level observations indicate association with HCC status and outcome but do not, by themselves, establish diagnostic or prognostic performance in an independent population.

### 3.2 Single‐cell transcriptome reveals enrichment of B4GALNT1 in hepatocyte populations and its association with ferroptosis/pyroptosis‐related phenotypes

Single‐cell RNA‐sequencing data were next used to map B4GALNT1 across the cellular compartments represented in HCC. UMAP clustering followed by canonical‐marker annotation resolved the principal parenchymal and immune populations in the dataset (Figure 1A and B). Comparison of normal‐adjacent and tumour specimens also revealed shifts in the relative abundance of hepatocyte‐associated and immune‐cell populations (Figure 1C).

**FIGURE 1:**
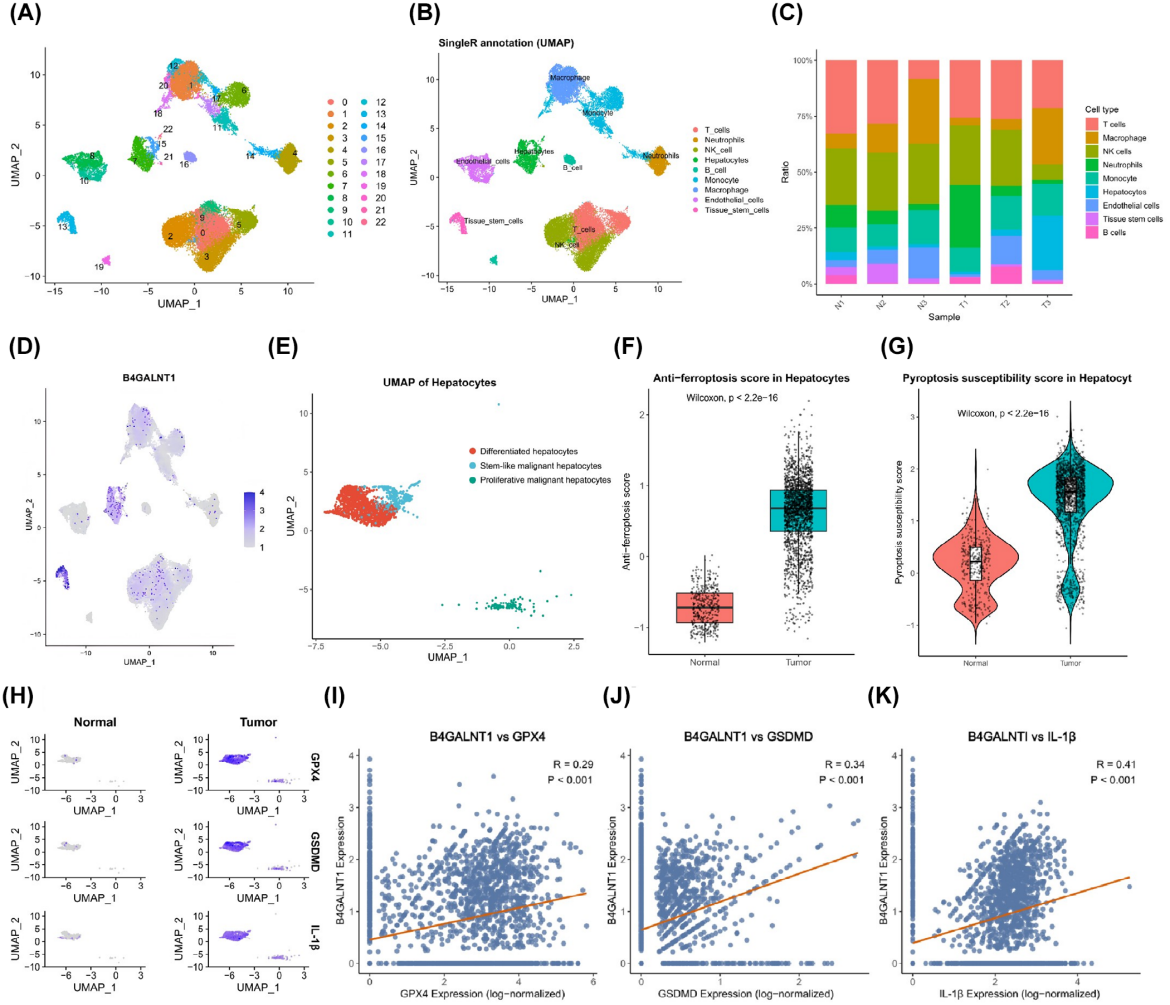
Single‐cell atlas identifies hepatocyte‐biased B4GALNT1 expression and coupled ferroptosis/pyroptosis signatures in HCC. (A–C) UMAP clustering, cell‐type annotation, and sample‐wise cell composition in the scRNA‐seq dataset; (D) B4GALNT1 FeaturePlot; (E) hepatocyte reclustering; (F, G) anti‐ferroptosis and pyroptosis‐susceptibility scores; (H) FeaturePlots of GPX4, GSDMD, and IL1B; (I–K) Spearman's correlations between B4GALNT1 and GPX4, GSDMD, and IL1B. The scRNA‐seq analysis included three adjacent‐normal and three tumour samples. Group comparisons used the Wilcoxon rank‐sum test, and gene–gene associations used Spearman's correlation. Statistical significance is denoted as *p < .05, **p < .01, ***p < .001, and ****p < .0001; ns, not significant.

B4GALNT1 expression localised mainly to hepatocyte‐associated regions of the atlas and was comparatively low in most non‐parenchymal populations (Figure 1D). Reanalysis of the hepatocyte compartment separated differentiated, stem‐like malignant, and proliferative malignant subclusters (Figure 1E), highlighting transcriptional heterogeneity within the hepatocyte lineage.

Tumour‐derived hepatocytes had higher anti‐ferroptosis and pyroptosis‐susceptibility scores than hepatocytes from adjacent‐normal tissue (Figure 1F and G). Because these metrics were derived from gene‐expression signatures, they were interpreted as pathway‐related transcriptional states rather than direct evidence that either death program had been executed.

Within hepatocytes, B4GALNT1 expression correlated positively with GPX4, GSDMD, and IL1B (*r* = .29, .34, and .41, respectively; all *p* < .001; Figure 1I–K). These single‐cell correlations support co‐variation among the measured genes but are not sufficient to infer direct regulatory relationships.

### 3.3 Local clinical cohort analysis supports the association between B4GALNT1 expression and sorafenib response in HCC

To extend the public dataset observations into a treatment‐annotated clinical setting, B4GALNT1 protein was evaluated in tumour specimens from 92 patients with HCC who received sorafenib between 2022 and 2023. Immunohistochemical H‐scores were used for quantification, and the cohort was divided at the median into B4GALNT1‐high and B4GALNT1‐low groups (46 patients each; Figure 2A).

**FIGURE 2:**
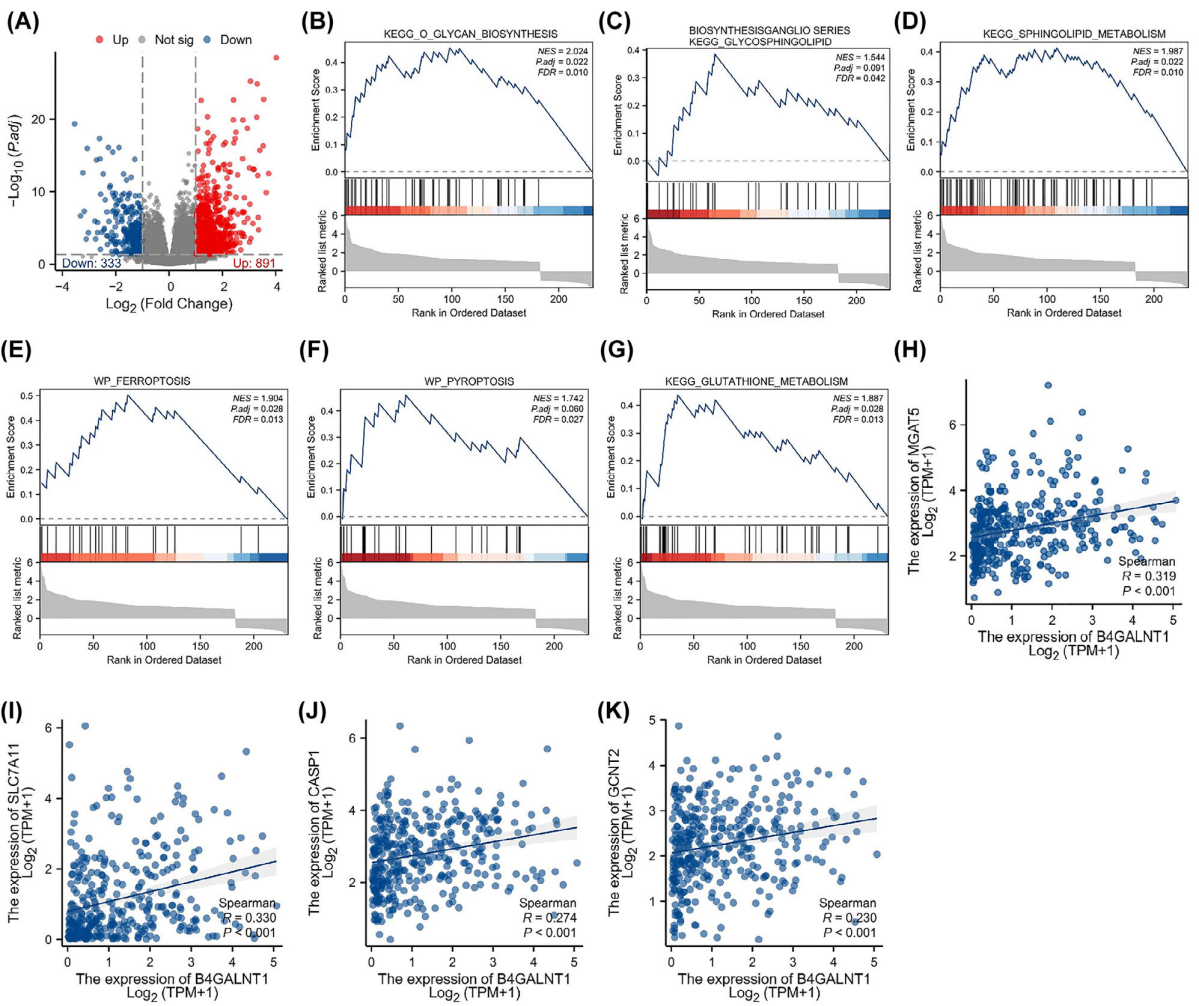
Local clinical cohort analysis supports the association between B4GALNT1 protein expression and sorafenib response in HCC. (A) Distribution of B4GALNT1 immunohistochemical H‐scores in the local sorafenib‐treated HCC cohort (*n* = 92); (B) H‐score comparison between disease‐control/responder and poor‐response/progressive‐disease groups; (C) treatment‐response composition according to B4GALNT1 expression status; (D) Kaplan–Meier analyses of progression‐free and overall survival; (E) multivariable Cox regression analysis. Continuous two‐group comparisons used the Mann–Whitney *U* test, categorical variables used the chi‐square test or Fisher's exact test as appropriate, survival curves used the log‐rank test, and hazard ratios were estimated by Cox proportional‐hazards regression. Statistical significance is denoted as *p < .05, **p < .01, ***p < .001, and ****p < .0001; ns, not significant.

B4GALNT1 H‐scores were greater in patients categorised as poor responders or with progressive disease than in patients with disease control (Figure 2B). Consistently, poor response/progression accounted for 60.9% of the B4GALNT1‐high group but 28.3% of the low‐expression group, whereas disease‐control status was more frequent in the low‐expression group (71.7% vs. 39.1%; Figure 2C). Kaplan–Meier curves showed shorter progression‐free and overall survival in the high‐expression group (log‐rank *p* = .0018 and *p* = .0015, respectively; Figure 2D). In multivariable Cox analysis, high B4GALNT1 expression remained associated with overall survival (HR = 1.97, 95% CI 1.07–3.61, *p* = .028; Figure 2E).

Thus, the local treatment‐annotated cohort independently supported an association between elevated B4GALNT1 protein and less favourable sorafenib response and clinical outcome. Given the retrospective design and cohort size, these findings were treated as exploratory clinical validation rather than as establishment of a predictive biomarker.

### 3.4 Pathway enrichment and GAT prioritisation link B4GALNT1 to glycosphingolipid and regulated cell‐death programs

We then positioned B4GALNT1 within broader HCC molecular programs by dividing TCGA‐LIHC tumours according to B4GALNT1 expression and examining differential expression, gene‐set enrichment, and gene‐level correlations.

The transcriptomic contrast between B4GALNT1‐high and B4GALNT1‐low tumours revealed widespread expression differences (Figure S2A), indicating that the two strata occupy distinct transcriptional states.

Pathway analysis showed enrichment of several glycan‐ and lipid‐associated programs in tumours with higher B4GALNT1. O‐glycan biosynthesis was significantly enriched (Figure S2B), while ganglio‐series glycosphingolipid biosynthesis and sphingolipid metabolism showed concordant enrichment trends (Figure S2C and D). These pathway‐level associations were not interpreted as evidence that B4GALNT1 directly catalyses protein O‐glycosylation.

Gene sets related to ferroptosis and pyroptosis also differed between the B4GALNT1 expression strata (Figure S2E and F), and glutathione‐metabolism genes were enriched in the high‐expression group (Figure S2G). Together, these findings linked B4GALNT1 abundance to redox‐ and regulated‐cell‐death‐associated transcriptional programs.

Within TCGA‐LIHC, B4GALNT1 showed positive Spearman's associations with GSDMD, SLC7A11, CASP1, and GCNT2 (Figure S2H–K). These analyses were used to identify genes that vary with B4GALNT1 expression and were not considered evidence of direct molecular regulation.

Taken together, the population‐level data placed B4GALNT1 within a transcriptional context that includes glycosphingolipid/sphingolipid metabolism, glutathione handling, and ferroptosis‐ and pyroptosis‐related programs.

To evaluate network‐level relationships, expression features were integrated with gene‐interaction topology in a graph attention network (GAT), and its classification performance was compared with support vector machine and random forest models (Figure 3A).

**FIGURE 3:**
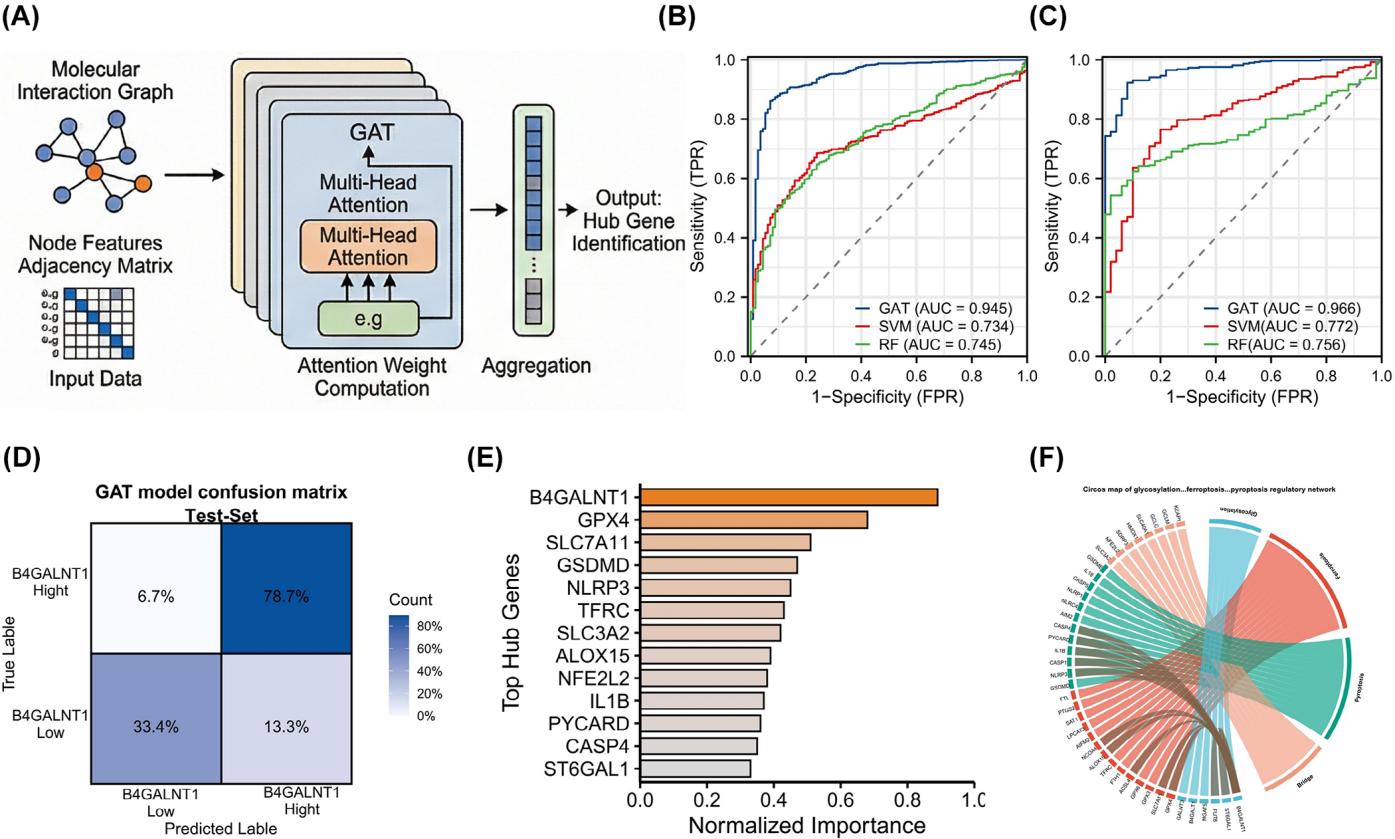
Graph attention network (GAT) prioritises B4GALNT1 as a cross‐module hub linking glycosylation, ferroptosis, and pyroptosis. (A) Schematic of the graph attention network (GAT) architecture; (B, C) ROC curves comparing GAT with support vector machine (SVM) and random forest (RF) models; (D) test‐set confusion matrix; (E) normalised feature‐importance ranking; (F) Circos visualisation of the integrated glycosylation–ferroptosis–pyroptosis network. Model construction, data splitting, normalisation, and performance evaluation are described in Methods.

The GAT produced a higher ROC performance than the SVM and RF comparators (Figure 3B and C). Its confusion matrix also showed good agreement between predicted and observed classes for B4GALNT1‐high versus B4GALNT1‐low status (Figure 3D), indicating stable discrimination in the analysed dataset.

Model‐derived feature rankings placed B4GALNT1 among the highest‐priority nodes and assigned it the largest normalised importance score. Other highly ranked genes included GPX4, SLC7A11, GSDMD, NLRP3, TFRC, SLC3A2, ALOX15, NFE2L2, IL1B, PYCARD, CASP4, and ST6GAL1 (Figure 3E). The functions represented by these genes span ferroptosis, lipid‐peroxide control, inflammasome signalling, and glycosylation.

A Circos representation was then used to visualise cross‐module connectivity in the integrated glycosylation–ferroptosis–pyroptosis network (Figure 3F). B4GALNT1 connected with glycosylation‐associated genes including ST6GAL1, GCNT2, and MGAT5, as well as with regulators of ferroptosis and inflammatory cell‐death signalling such as GPX4, SLC7A11, TFRC, ALOX15, GSDMD, NLRP3, CASP1, CASP4, IL1B, and PYCARD.

Overall, the network analysis prioritised B4GALNT1 as a cross‐module node associated with glycosylation, ferroptosis, and pyroptosis‐related gene programs in HCC.

### 3.5 B4GALNT1 is significantly upregulated in the acquired‐resistant Huh7‐SR cell line

We next compared parental Huh7 cells with the long‐term sorafenib‐resistant derivative Huh7‐SR to determine whether acquisition of resistance was accompanied by altered B4GALNT1 expression.

B4GALNT1 protein abundance was higher in Huh7‐SR than in Huh7 cells (Figure S3A), and RT‐qPCR showed a concordant increase in transcript abundance (Figure S3B). Relative to parental cells, Huh7‐SR cells retained a higher JC‐1 red/green ratio and ATP content, released less LDH after sorafenib exposure, and exhibited a larger resistance index (Figure S3C–F). During continued drug exposure, Huh7‐SR cells also maintained viability more effectively than Huh7 cells (Figure S3G).

These measurements confirmed the acquired‐resistant phenotype of Huh7‐SR and showed that increased B4GALNT1 expression accompanied the resistant state.

### 3.6 B4GALNT1 upregulation drives resistance, whereas KO restores sorafenib sensitivity

Stable gain‐ and loss‐of‐function models were used to test how B4GALNT1 abundance influences the response to sorafenib (Figure S4A). Western blotting and RT‐qPCR verified increased expression in Oe‐B4GALNT1 cells and effective depletion in KO‐B4GALNT1 cells (Figure S4B and C).

Immunofluorescence showed the expected increase in B4GALNT1 signal after overexpression and decrease after knockout (Figure S4D). Morphologically, Oe‐B4GALNT1 cultures appeared denser, whereas KO‐B4GALNT1 cultures were less compact (Figure S4E). Under drug‐free conditions, proliferation was modestly higher after B4GALNT1 overexpression and lower after knockout (Figure S4F).

Sorafenib‐response measurements showed reciprocal effects of B4GALNT1 manipulation. Oe‐B4GALNT1 shifted the dose–response curve toward lower sensitivity, while KO‐B4GALNT1 increased sensitivity (Figure 4A). Colony formation and EdU incorporation were greater in Oe‐B4GALNT1 cells and lower in KO cells during sorafenib treatment (Figure 4B and C). Annexin V/PI analysis showed the largest drug‐associated death fraction after knockout and the smallest after overexpression (Figure 4D). Time‐course measurements likewise showed sustained protection in Oe‐B4GALNT1 cells and the greatest viability loss in KO cells (Figure 4E). Relative IC_50_ values followed the same ordering (Figure 4F).

**FIGURE 4:**
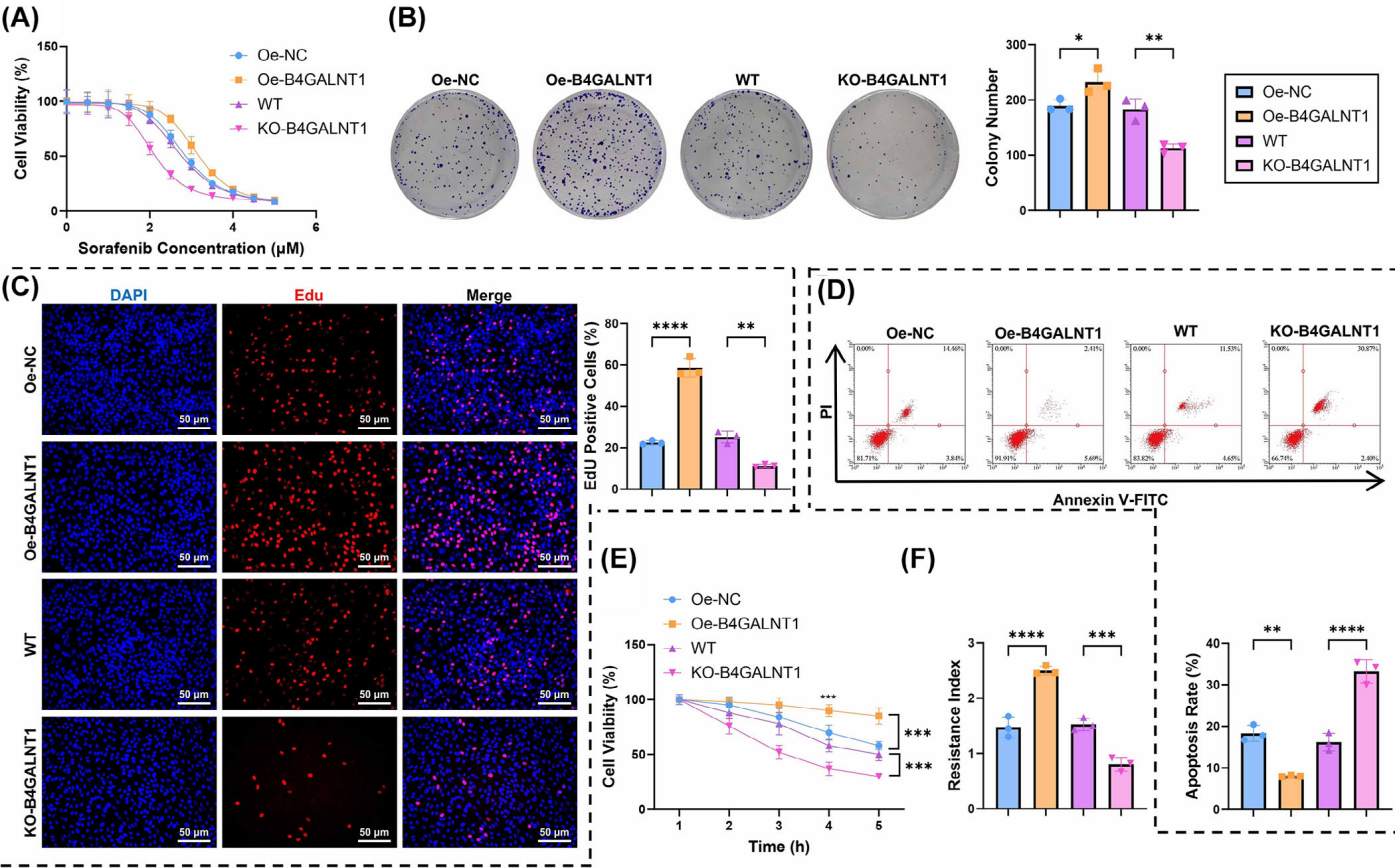
B4GALNT1 bidirectionally modulates sorafenib sensitivity in vitro. (A) Sorafenib dose–response curves with IC_50_ estimation; (B) colony‐formation assay; (C) EdU incorporation assay; (D) Annexin V/PI flow‐cytometric analysis; (E) time‐course cell‐viability assay; (F) resistance index. Cell experiments were performed in three independent biological replicates. Dose–response curves were fitted by nonlinear regression; panels B–D and F were analysed by one‐way ANOVA followed by Tukey's multiple‐comparisons test, and panel E was analysed by two‐way ANOVA followed by Sidak's multiple‐comparisons test. Statistical significance is denoted as *p < .05, **p < .01, ***p < .001, and ****p < .0001; ns, not significant.

Across these functional assays, increasing B4GALNT1 reduced sorafenib responsiveness, whereas B4GALNT1 loss increased drug sensitivity.

### 3.7 B4GALNT1 suppresses ferroptotic injury while promoting restricted caspase‐1/GSDMD signalling

Multiple biochemical and imaging endpoints were used to define the ferroptosis‐associated phenotype after B4GALNT1 manipulation. Oe‐B4GALNT1 cells showed lower C11‐BODIPY oxidation (Figure 5A) and less FerroOrange‐detectable labile Fe^2^^+^ (Figure 5B and left subpanel of Figure 5C). The GSH/GSSG ratio was higher in the overexpression condition (right subpanel of Figure 5C), consistent with a more reducing intracellular environment. MDA and DCF‐DA measurements likewise showed reduced lipid‐peroxidation products and total ROS (Figure 5D and E). Transmission electron microscopy documented corresponding mitochondrial changes (Figure 5F). GPX4 and SLC7A11 protein abundance increased with B4GALNT1 overexpression and declined after knockout (Figure 5G), while PI staining showed fewer membrane‐compromised cells under the tested stress condition (Figure 5H). Figure 5I summarises the integrated phenotype.

**FIGURE 5:**
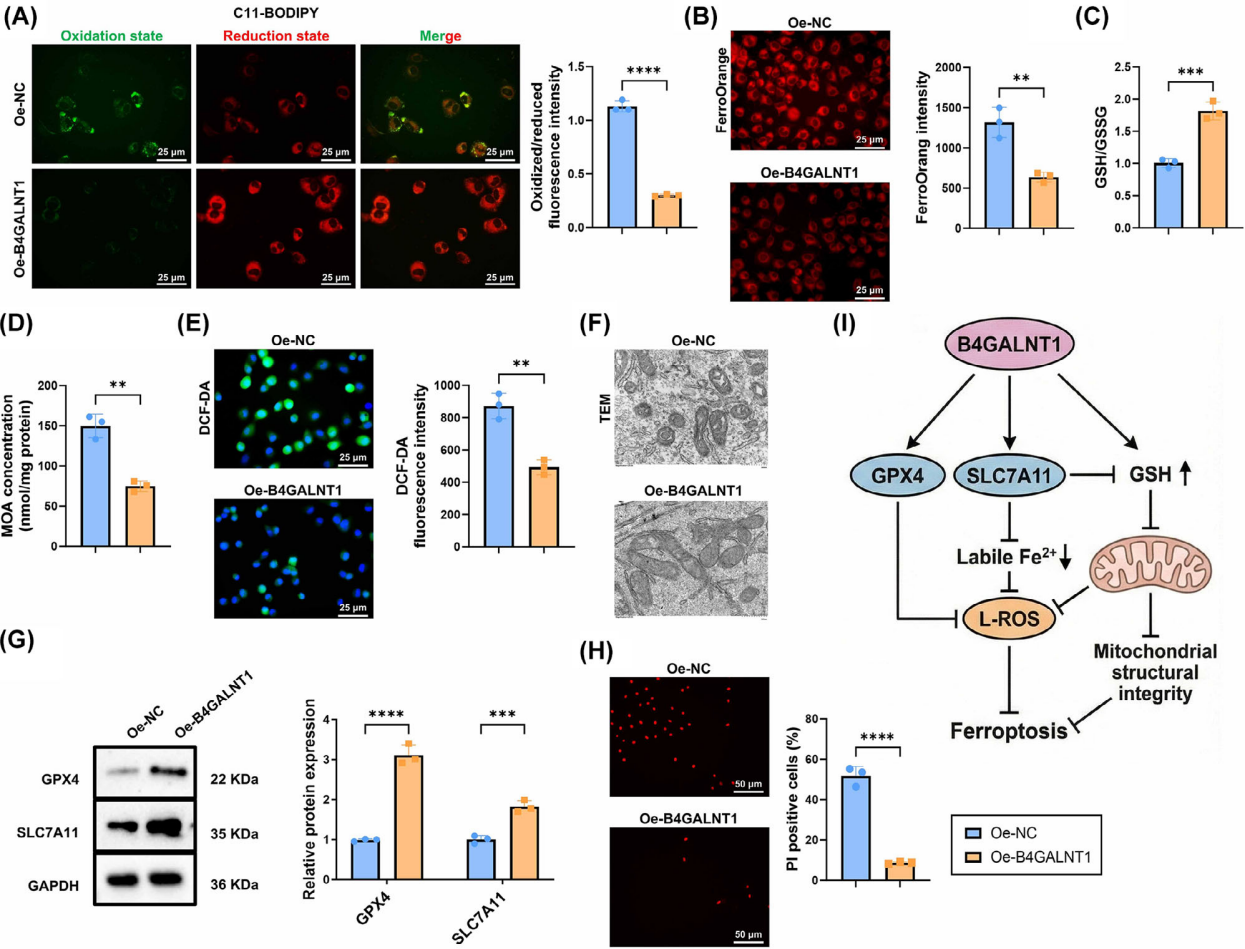
B4GALNT1 overexpression suppresses ferroptosis through multi‐node antioxidant reinforcement. (A) C11‐BODIPY analysis of lipid peroxidation; (B) FerroOrange imaging of labile Fe^2^^+^; (C) FerroOrange quantification and GSH/GSSG measurements; (D) MDA assay; (E) DCF‐DA analysis of cellular ROS; (F) transmission electron microscopy of mitochondrial ultrastructure; (G) Western blot analysis of GPX4 and SLC7A11; (H) PI staining and quantification; (I) schematic summary. Cell experiments were performed in three independent biological replicates. Quantitative comparisons in panels A–E, G, and H were analysed by one‐way ANOVA followed by Tukey's multiple‐comparisons test. Scale bars are indicated in the corresponding images. Statistical significance is denoted as *p < .05, **p < .01, ***p < .001, and ****p < .0001; ns, not significant.

At the same time, cleaved caspase‐1 and GSDMD‐N increased with B4GALNT1 overexpression (Figure 6A and B), together with greater IL‐1β and IL‐18 secretion (Figure 6C). Annexin V/PI was used as a general death‐status measurement rather than as a pyroptosis‐specific classifier (Figure 6D). Immunofluorescence showed increased membrane‐associated GSDMD‐N in the overexpression condition (Figure 6E).

**FIGURE 6:**
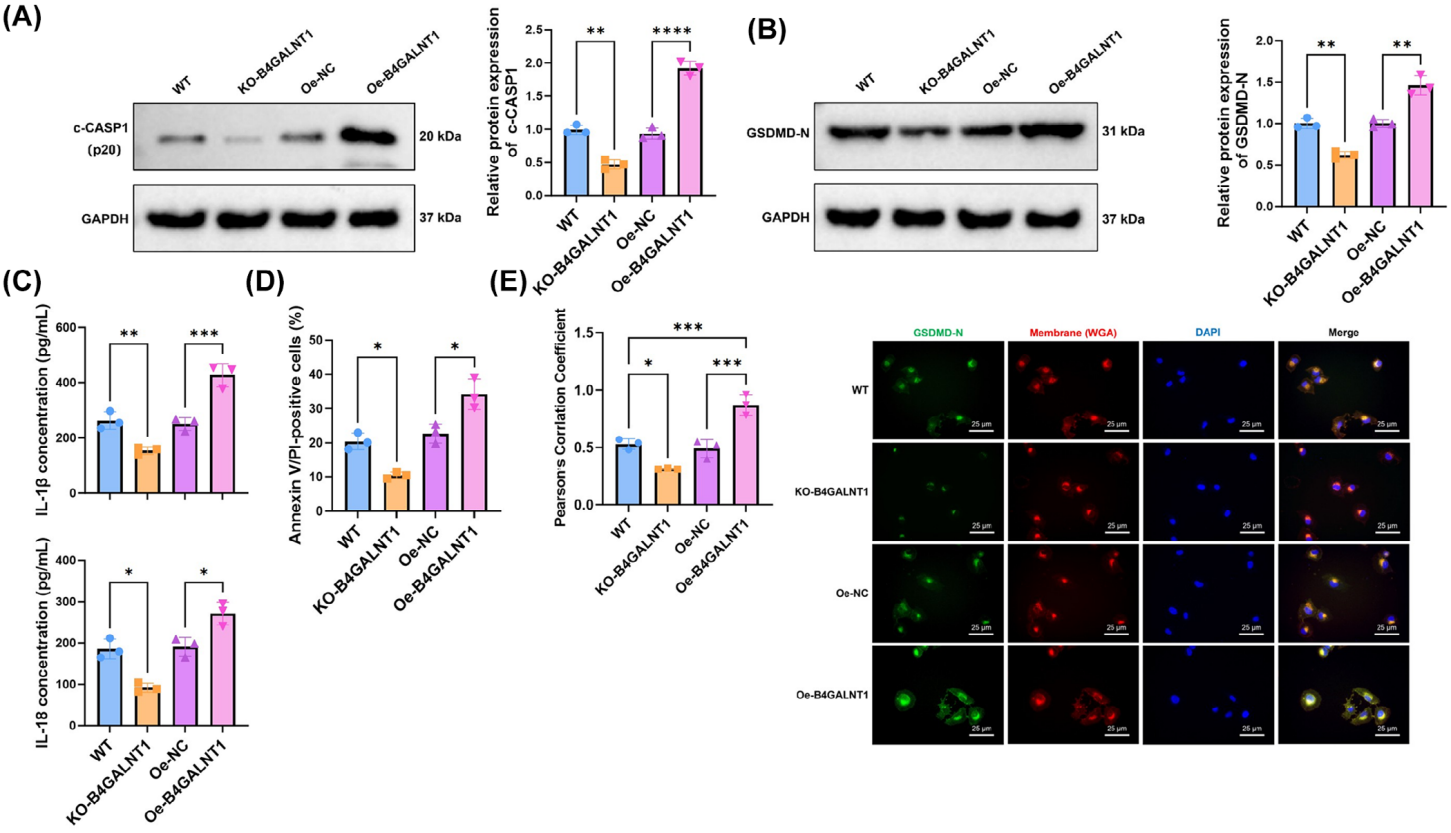
B4GALNT1 promotes caspase‐1/GSDMD signalling with membrane‐localised GSDMD‐N while maintaining viability. (A) Western blot analysis of cleaved CASP1; (B) Western blot analysis of GSDMD‐N; (C) ELISA measurement of IL‐1β and IL‐18; (D) Annexin V/PI analysis; (E) GSDMD‐N/WGA/DAPI immunofluorescence with quantitative membrane‐localisation analysis. Cell experiments were performed in three independent biological replicates. Quantitative comparisons were analysed by one‐way ANOVA followed by Tukey's multiple‐comparisons test. Statistical significance is denoted as *p < .05, **p < .01, ***p < .001, and ****p < .0001; ns, not significant.

Taken together, B4GALNT1‐high cells displayed reduced ferroptosis‐associated injury while simultaneously showing stronger caspase‐1/GSDMD signalling and cytokine release. The membrane‐permeability kinetics remained restricted and did not indicate widespread terminal lysis.

### 3.8 B4GALNT1 drives ganglioside metabolic reprogramming and promotes lipid‐raft remodelling enriched with GM1/GM2

We next examined whether B4GALNT1 alters ganglioside composition and membrane‐domain organisation by comparing WT, Oe‐NC, Oe‐B4GALNT1, and KO‐B4GALNT1 cells.

TLC resolved distinct GM3, GM2, and GM1 patterns across the four groups (Figure S5A). Oe‐B4GALNT1 increased GM2 and GM1 band intensity, whereas knockout reduced these signals; WT and Oe‐NC were similar. Densitometric analysis confirmed the same pattern (Figure S5B). GM2 immunofluorescence increased after overexpression and decreased after knockout (Figure S5C and D), and GM1 staining changed in the same direction (Figure S5E and F).

Targeted quantification further showed greater GM2, GM1, and GD2 abundance in Oe‐B4GALNT1 cells and lower values after knockout (Figure S5G). In detergent‐resistant membrane fractions, GM1 enrichment was greatest with B4GALNT1 overexpression and lowest after knockout, with WT and Oe‐NC at intermediate levels (Figure S5H). These data demonstrate changes in ganglioside composition and raft‐associated GM1 enrichment but do not constitute a direct metabolic‐flux measurement.

Orthogonal membrane‐domain assays provided additional support for the B4GALNT1–ganglioside–raft relationship. Acute MβCD exposure reduced DRM‐associated GM1 enrichment across genetic backgrounds (Figure S5I), whereas cholesterol replenishment partially recovered the phenotype (Figure S5J). DRM fractions showed corresponding changes in Flotillin‐1, Caveolin‐1, and CTxB‐detected GM1 (Figure S5K). In a separate ferroptosis‐focused module, erastin increased PI‐positive cells and ferrostatin‐1 blunted this effect (Figure S5L and M).

Thus, higher B4GALNT1 shifted ganglioside abundance toward GM2/GM1/GD2 and increased the GM1‐enriched raft‐associated fraction, whereas B4GALNT1 loss caused the reverse pattern.

### 3.9 B4GALNT1 perturbation is associated with secondary changes in cell surface glycan readouts

To determine whether ganglioside remodelling was accompanied by broader changes in surface glycan readouts, lectin‐based measurements and O‐glycoproteomic profiles were compared among WT, KO‐B4GALNT1, Oe‐NC, and Oe‐B4GALNT1 cells.

VVA and SBA immunofluorescence was stronger and more continuous at the cell surface after B4GALNT1 overexpression and weaker after knockout (Figure 7A and B), corresponding to altered O‐GalNAc‐ and T‐antigen‐reactive lectin binding. Flow‐cytometric histograms and mean fluorescence intensity measurements showed increased binding of VVA, SBA, and RCA‐I in Oe‐B4GALNT1 cells and lower binding after knockout (Figure 7C and D).

**FIGURE 7:**
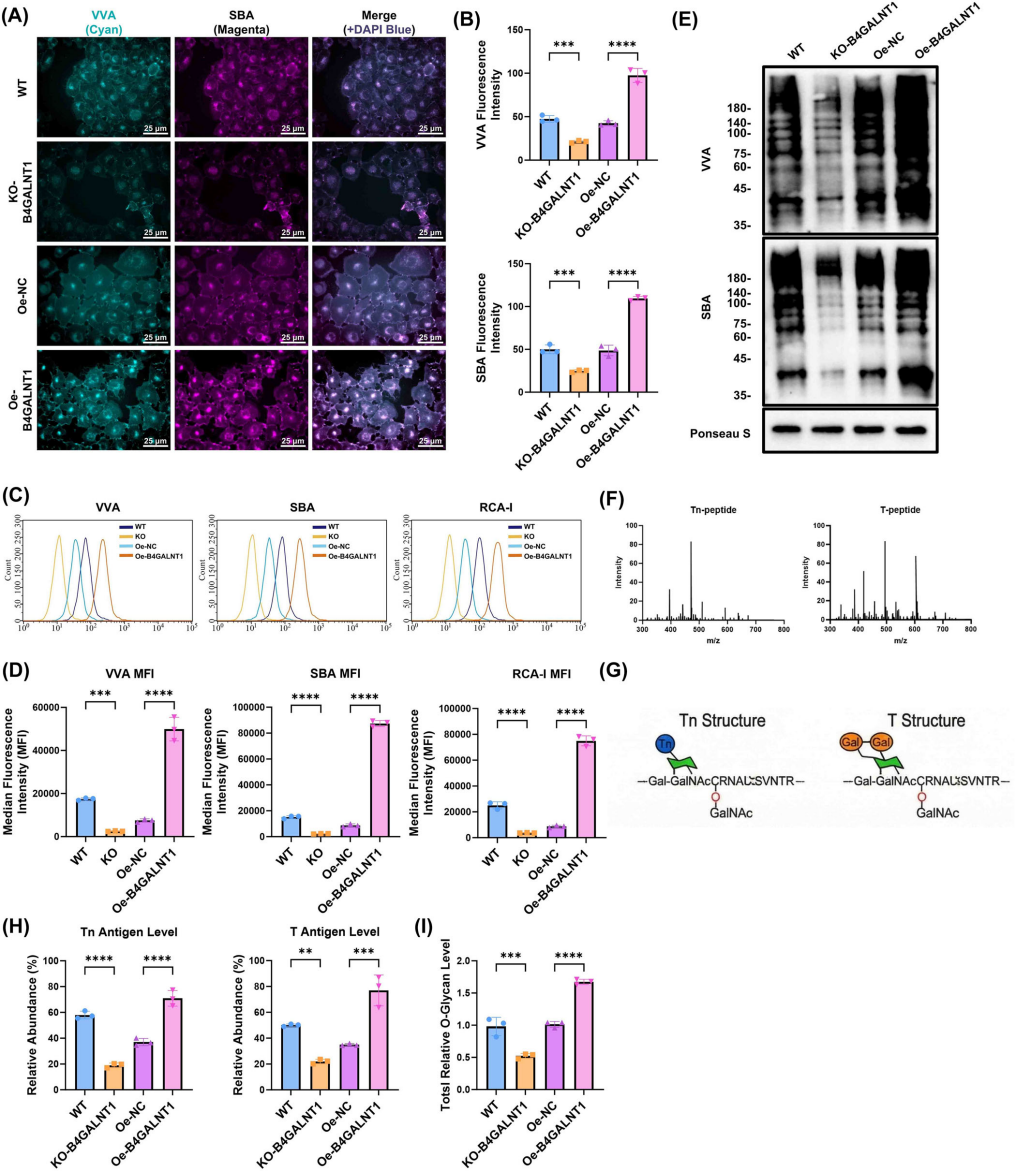
B4GALNT1 intervention increases cell surface O‐GalNAc/T‐antigen exposure and lectin binding while overall interpretation remains indirect. (A, B) VVA and SBA lectin immunofluorescence with quantitative analysis; (C, D) flow‐cytometric histograms and mean fluorescence intensity measurements for VVA, SBA, and RCA‐I; (E) VVA/SBA lectin blot with Ponceau S total protein staining; (F, G) representative O‐glycopeptide MS/MS spectra and glycan annotations; (H, I) relative O‐glycan abundance measurements. Cell experiments were performed in three independent biological replicates. Multi‐group quantitative data were analysed by one‐way ANOVA followed by Tukey's multiple‐comparisons test. Statistical significance is denoted as *p < .05, **p < .01, ***p < .001, and ****p < .0001; ns, not significant.

Lectin blots similarly showed stronger VVA‐ and SBA‐reactive bands across several molecular‐weight regions after B4GALNT1 overexpression and weaker signals after knockout, while Ponceau S staining indicated comparable total protein loading (Figure 7E).

Representative O‐glycopeptide MS/MS spectra and Tn/T annotations are presented in Figure 7F and G. Quantitative O‐glycoproteomics showed higher relative abundance of Tn/T‐antigen‐associated structures and total O‐glycan signal in Oe‐B4GALNT1 cells and lower abundance in KO‐B4GALNT1 cells (Figure 7H and I). Because B4GALNT1 is a ganglioside biosynthetic enzyme, these glycan changes were interpreted as secondary consequences of altered ganglioside/membrane organisation rather than as direct protein O‐GalNAc catalysis by B4GALNT1.

### 3.10 GM1 supplementation partially reverses ferroptosis‐associated changes and restores caspase‐1/GSDMD‐related signalling

After establishing B4GALNT1‐dependent changes in gangliosides and raft organisation, exogenous GM1 was used to test whether the cell‐death‐associated phenotype of B4GALNT1‐deficient cells could be partially reversed. The Huh7‐SR‐derived Vector, Oe‐B4GALNT1, KO‐B4GALNT1, and KO‐B4GALNT1 + GM1 groups used for these assays are summarised in Figure 8A.

**FIGURE 8:**
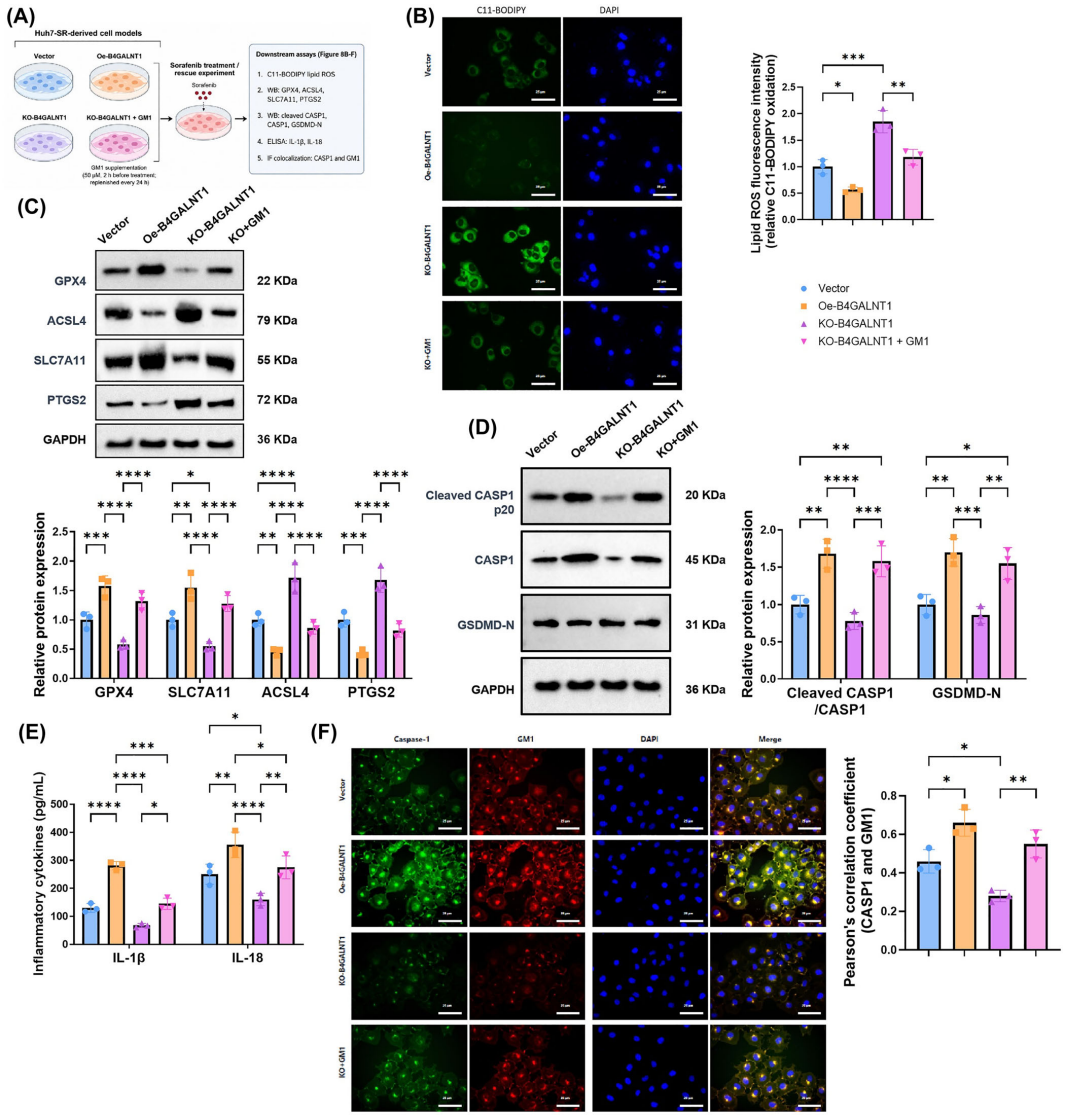
GM1 supplementation partially rescues ferroptosis‐ and pyroptosis‐associated alterations in Huh7‐SR‐derived cell models. (A) Experimental design using Huh7‐SR‐derived Vector, Oe‐B4GALNT1, KO‐B4GALNT1, and KO‐B4GALNT1 + GM1 cells; (B) C11‐BODIPY analysis; (C) Western blot analysis of GPX4, ACSL4, SLC7A11, and PTGS2; (D) Western blot analysis of cleaved CASP1/CASP1 and GSDMD‐N; (E) ELISA measurement of IL‐1β and IL‐18; (F) CASP1‐GM1 immunofluorescence colocalisation with Pearson's correlation analysis. Experiments were performed in three independent biological replicates. Quantitative comparisons were analysed by one‐way ANOVA followed by Tukey's multiple‐comparisons test. Statistical significance is denoted as *p < .05, **p < .01, ***p < .001, and ****p < .0001; ns, not significant.

C11‐BODIPY showed the lowest lipid ROS in Oe‐B4GALNT1 cells and the highest signal after B4GALNT1 knockout; GM1 addition reduced the knockout‐associated increase (Figure 8B). GPX4 and SLC7A11 were increased with B4GALNT1 overexpression and decreased after knockout, whereas GM1 partially restored both proteins; ACSL4 and PTGS2 changed in the reciprocal direction (Figure 8C).

In the same rescue series, cleaved CASP1/CASP1 and GSDMD‐N increased after B4GALNT1 overexpression and fell after knockout, with partial restoration following GM1 supplementation (Figure 8D). IL‐1β and IL‐18 secretion followed a similar pattern (Figure 8E). CASP1–GM1 colocalisation was strongest in Oe‐B4GALNT1 cells, weakest in KO cells, and partly restored by GM1 in the knockout background (Figure 8F).

Overall, exogenous GM1 partially reversed the ferroptosis‐associated changes and restored several caspase‐1/GSDMD‐related readouts in B4GALNT1‐deficient cells, supporting a functional contribution of ganglioside‐dependent membrane organisation.

### 3.11 GM1 supplementation partially restores sorafenib tolerance in B4GALNT1‐deficient cells

To assess whether GM1 also affected drug tolerance in B4GALNT1‐deficient cells, KO‐B4GALNT1 + Sorafenib and KO‐B4GALNT1 + GM1 + Sorafenib groups were compared using viability, cell‐death, dose–response, colony‐formation, and redox measurements (Figure S6A–F).

CCK‐8 measurements showed higher viability after GM1 rescue in KO cells (Figure S6B). Annexin V/PI flow cytometry indicated a lower drug‐associated death fraction after GM1 supplementation (Figure S6C). Dose–response analysis showed a higher IC_50_ after GM1 addition (Figure S6D), and colony growth under prolonged drug exposure was also increased (Figure S6E). Cellular GSH measurements changed in the same direction as the functional recovery after GM1 supplementation (Figure S6F).

Together, these findings indicate that GM1 partially restores sorafenib tolerance after B4GALNT1 loss, consistent with a functional role for ganglioside‐dependent membrane organisation.

### 3.12 Cross‐model, genetic specificity, and pharmacological validation

Cross‐model validation in HepG2, a hepatoblastoma‐derived liver‐tumour model, and MHCC97H, an HCC model, together with their sorafenib‐resistant derivatives, reproduced the principal directional changes associated with B4GALNT1 manipulation in sorafenib response, ferroptosis‐associated readouts, and caspase‐1/GSDMD‐related signalling (Figure S7A–E).

To assess guide and clone specificity, an independent KO‐2 clone generated with a second sgRNA targeting a distinct B4GALNT1 exon was examined in both Huh7 and Huh7‐SR cells. WT and sgNC controls showed comparable profiles, whereas KO‐2 reduced B4GALNT1, GPX4, and SLC7A11 protein abundance and lowered GPX4/SLC7A11 mRNA (Figure S8A and B). Cleaved CASP1, GSDMD‐N, and IL‐1β/IL‐18 secretion were likewise decreased (Figure S8C and D). The 72‐h sorafenib dose–response curve remained right‐shifted in Huh7‐SR relative to Huh7, while KO‐2 shifted the response leftward within each background (Figure S8E). KO‐2 also increased MDA, DCF‐DA‐detected ROS, PI‐positive cells, and MitoSOX‐detected mitochondrial ROS (Figure S8F–J).

Functional specificity was further examined in Huh7‐SR cells using time‐resolved membrane‐permeability and pharmacological rescue assays. PI/YO‐PRO‐1 uptake kinetics, LDH release, cleaved caspase‐3 measurements, and caspase‐inhibitor experiments characterised restricted caspase‐1/GSDMD‐associated membrane permeabilisation (Figure S9A–D). Oe‐B4GALNT1 cells reached the first detectable YO‐PRO‐1‐positive event earlier, whereas KO

‐B4GALNT1 cells reached this endpoint later (Figure S9I); a measurable fraction of YO‐PRO‐1‐positive cells continued to exclude PI at 24 h (Figure S9J). In parallel ferrostatin‐1 rescue modules, KO‐B4GALNT1 + Sorafenib produced higher lipid ROS and LDH release and lower viability and ATP, whereas ferrostatin‐1 reversed these changes. Oe‐B4GALNT1 + Sorafenib showed the opposite profile, which was maintained or further improved by ferrostatin‐1 (Figure S9E–H).

Finally, catalytic dependence was tested by re‐expressing wild‐type B4GALNT1 or the D358A catalytic‐loss variant in KO cells. Wild‐type rescue increased GM1 enrichment and restored GPX4 and SLC7A11 abundance, whereas D358A produced only limited recovery (Figure S10A and B). Cleaved CASP1 and GSDMD‐N showed the same pattern, with robust restoration by wild‐type B4GALNT1 but little change after D358A rescue (Figure S10C). Sorafenib resistance, expressed as relative IC50, increased after wild‐type rescue but changed only weakly with D358A (Figure S10D).

### 3.13 B4GALNT1/GM1 modulation alters in vivo tumour growth and cell‐death‐associated readouts

The in vivo relevance of B4GALNT1/GM1 modulation was examined in subcutaneous xenografts established from Huh7‐SR‐derived Vector, Oe‐B4GALNT1, KO‐B4GALNT1, and KO‐B4GALNT1 + GM1 cells; the study design is shown in Figure 9A.

**FIGURE 9:**
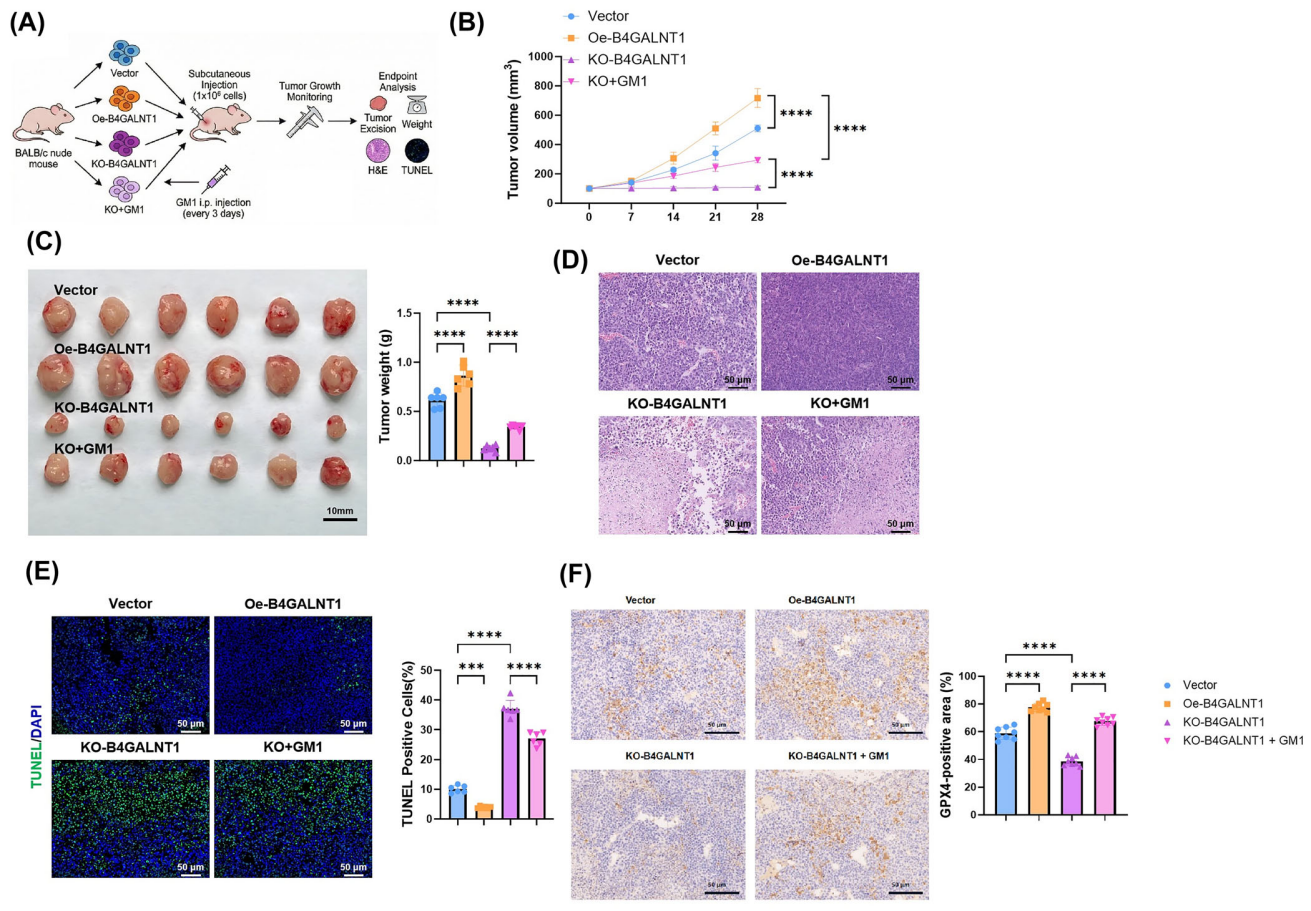
In vivo tumour growth, histology, TUNEL positivity, and GPX4 expression reflect B4GALNT1‐driven phenotypes and partial GM1 rescue. (A) Subcutaneous‐xenograft experimental design using Huh7‐SR‐derived groups; (B) tumour‐volume measurements over time; (C) endpoint tumour images and tumour‐weight quantification; (D) H&E staining; (E) TUNEL staining and quantification; (F) GPX4 immunohistochemistry and quantification. Animal experiments included six mice per group. Tumour‐volume data were analysed by repeated‐measures ANOVA with post hoc comparisons, and endpoint multi‐group measurements used one‐way ANOVA with Tukey's multiple‐comparisons test. Scale bars are shown in the figure. Statistical significance is denoted as *p < .05, **p < .01, ***p < .001, and ****p < .0001; ns, not significant.

Tumours grew fastest in the Oe‐B4GALNT1 group and slowest after B4GALNT1 knockout, while GM1 supplementation partially recovered growth in the KO group (Figure 9B). Endpoint tumour images and tumour weights showed the same ordering (Figure 9C). H&E sections showed denser tumour architecture in Oe‐B4GALNT1 xenografts and more degenerative/necrotic changes after knockout (Figure 9D). TUNEL positivity was greatest in KO‐B4GALNT1 tumours and lowest after overexpression, with an intermediate signal after GM1 rescue (Figure 9E). GPX4 immunohistochemistry showed the reciprocal distribution, with the highest positive area in Oe‐B4GALNT1 tumours and the lowest in KO tumours (Figure 9F).

Additional tumour‐tissue measurements showed the inverse pattern for 4‐HNE, which was highest after knockout and lower in Oe‐B4GALNT1 tumours, with partial reduction after GM1 supplementation (Figure 10A). GPX4 staining again followed the reciprocal direction (Figure 10B). Cleaved CASP1 and GSDMD‐N immunohistochemistry was stronger in Oe‐B4GALNT1 tumours and weaker after knockout, with partial restoration by GM1 (Figure 10C and D).

**FIGURE 10:**
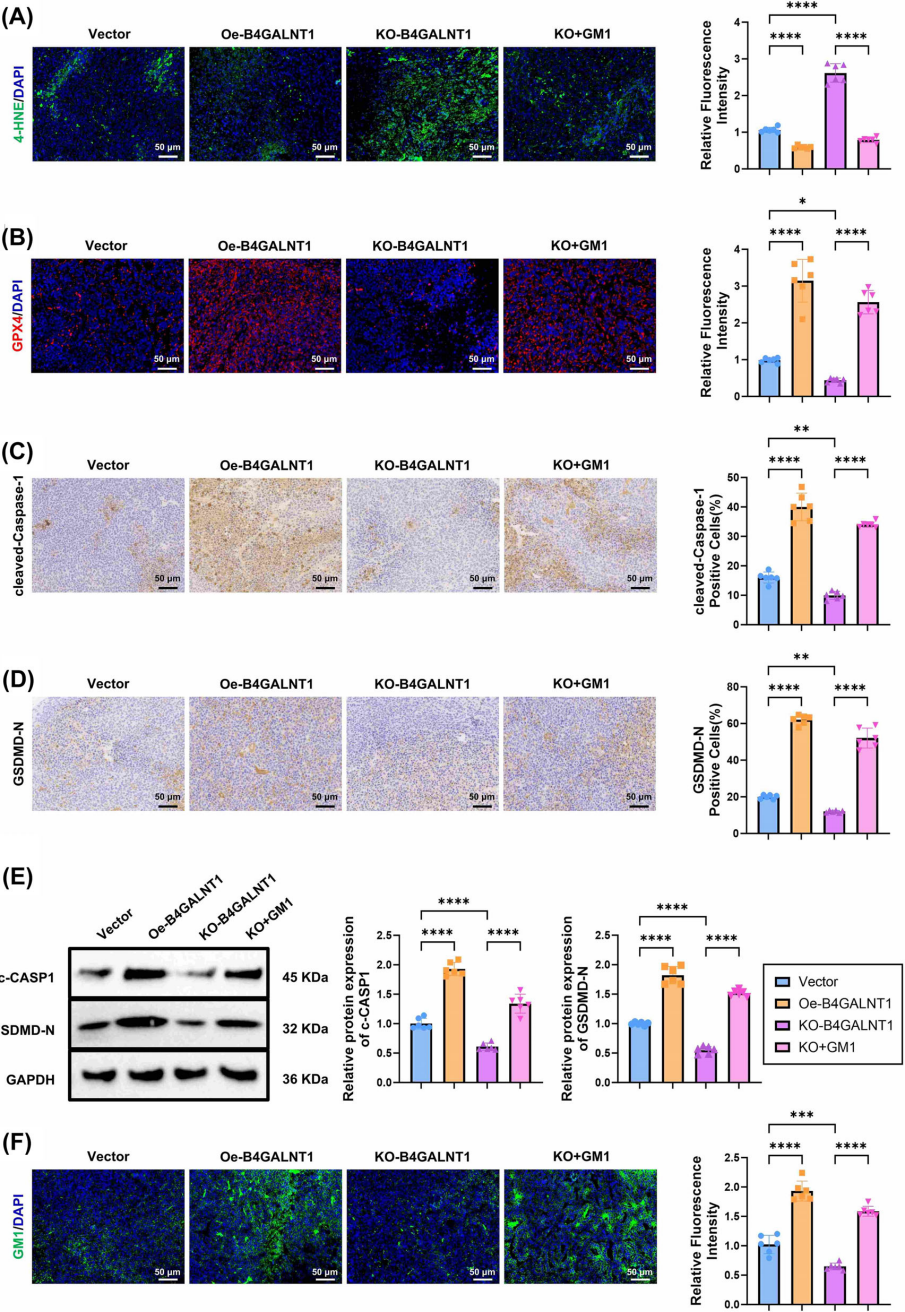
In vivo molecular readouts reveal oxidative damage and pyroptosis‐related alterations associated with B4GALNT1 and GM1. (A) Tumour 4‐HNE staining and quantification; (B) GPX4 staining and quantification; (C, D) cleaved CASP1 and GSDMD‐N immunohistochemistry with quantitative analysis; (E) Western blot analysis of cleaved CASP1/CASP1 and GSDMD‐N in tumour tissue; (F) GM1 staining and quantification. Animal experiments included six mice per group. Quantitative comparisons were analysed by one‐way ANOVA followed by Tukey's multiple‐comparisons test. Scale bars are indicated in the figure. Statistical significance is denoted as *p < .05, **p < .01, ***p < .001, and ****p < .0001; ns, not significant.

Western blotting confirmed concordant changes in cleaved CASP1/CASP1 and GSDMD‐N (Figure 10E). GM1 staining was highest in Oe‐B4GALNT1 tumours, lowest after knockout, and partially recovered after GM1 supplementation (Figure 10F).

The xenograft results therefore paralleled the cell‐culture findings: higher B4GALNT1 was associated with lower ferroptosis‐related oxidative injury and stronger caspase‐1/GSDMD‐associated signalling, whereas knockout reversed these features and GM1 provided partial rescue.

## 4 DISCUSSION

The present study supports a model in which B4GALNT1 contributes to sorafenib resistance by reshaping ganglioside composition and membrane microdomains rather than by acting solely through a conventional proliferation pathway. Previous HCC studies have associated B4GALNT1 with aggressive tumour traits, including stemness, invasion, progression, integrin‐linked signalling, and poorer prognosis.^25^, ^26^ Our data extend this context by linking B4GALNT1 to a membrane‐centred program in which GM1/GM2‐rich raft organisation accompanies greater ferroptosis resistance and restricted caspase‐1/GSDMD‐associated signalling. Because these conclusions are supported by gain‐ and loss‐of‐function models, an independent second sgRNA, GM1 rescue, a catalytic‐loss D358A comparison, ferroptosis‐directed pharmacology, and xenograft experiments, the study moves beyond a purely correlative association and provides a functional framework for how B4GALNT1 may participate in acquired sorafenib tolerance.

Several complementary data layers were used to reach this conclusion. TCGA‐LIHC provided population‐level associations with tumour status, stage, and outcome, whereas single‐cell transcriptomics localised B4GALNT1 expression mainly to hepatocyte‐associated compartments and linked it to anti‐ferroptosis and pyroptosis‐related transcriptional signatures within hepatocytes. The local treatment‐annotated cohort added protein‐level clinical context by showing that higher B4GALNT1 expression was associated with poorer sorafenib response and less favourable survival. These datasets differ in scale and biological resolution, and each has limitations: public single‐cell datasets can be influenced by sample selection and batch structure, while the local cohort is retrospective and modest in size. The experimental models therefore served as an independent functional layer. Huh7/Huh7‐SR cells captured acquired drug adaptation, additional liver‐tumour models tested directional reproducibility, and xenografts assessed whether the cell‐culture phenotype persisted in vivo. Together, these elements create a cross‐supporting evidence chain while avoiding the assumption that association in one data type establishes causality.

The GAT analysis was used primarily as a prioritisation and integration tool. Unlike simple differential‐expression or enrichment analyses, a graph‐based model can incorporate both expression features and predefined relationships among genes, allowing attention weights to highlight nodes that bridge functional modules. B4GALNT1 emerged as a high‐priority node connecting glycosylation‐related genes with regulators of ferroptosis and inflammatory cell‐death signalling. Benchmarking against SVM and RF reduced reliance on a single modelling approach, but the network remains constrained by the curated candidate set and graph topology. Accordingly, the GAT results are most appropriately interpreted as hypothesis‐generating rather than as an independent demonstration of mechanism. The mechanistic claims in this study instead depend on perturbation experiments and rescue analyses that directly tested the prioritised axis.

Mechanistically, B4GALNT1 should be viewed as a ganglioside biosynthetic enzyme rather than as a protein O‐GalNAc transferase. Its established activity is the β1,4 transfer of GalNAc within the GM3/GD3‐to‐GM2/GD2 branch; therefore, the primary biochemical phenotype observed here is altered ganglioside composition and GM1‐enriched membrane organisation. The accompanying changes in VVA/SBA binding and O‐glycoproteomic profiles are more plausibly secondary consequences of altered membrane and metabolic organisation than direct protein glycosylation by B4GALNT1. This interpretation is supported by the TLC/LC‐MS, DRM, MβCD/cholesterol, and GM1‐rescue experiments. The D358A rescue comparison further indicates that catalytic competence is important for recovery of GM1 enrichment, ferroptosis‐associated proteins, caspase‐1/GSDMD readouts, and sorafenib resistance. At the same time, exogenous GM1 is not specific to B4GALNT1 and can influence membrane organisation broadly; its rescue effect should therefore be considered supportive rather than definitive evidence for a unique downstream mediator.

A second feature of the model is the coexistence of ferroptosis resistance with increased caspase‐1/GSDMD‐associated signalling. Reduced lipid ROS, labile Fe^2^^+^, and oxidative‐damage markers, together with higher GPX4/SLC7A11 and ferrostatin‐1 rescue, support a ferroptosis‐specific protective component. In parallel, cleaved CASP1, GSDMD‐N, cytokine release, membrane‐associated GSDMD‐N, and caspase‐inhibitor responses indicate engagement of the pyroptotic machinery. However, LDH release remained limited and a substantial fraction of YO‐PRO‐1‐positive cells still excluded PI at 24 h. These findings are therefore more consistent with restricted or partially executed membrane permeabilisation than with uniform, fully lytic pyroptosis. Because the same individual permeabilised cells were not followed continuously to their ultimate fate, the data do not establish durable single‐cell survival and should not be interpreted as formal lineage‐resolved fate tracking.

The translational implications should also be interpreted cautiously. In the local cohort, higher B4GALNT1 protein expression was associated with poorer sorafenib response and less favourable outcome, extending the TCGA survival association into a treatment‐annotated setting. Previous work has likewise connected B4GALNT1 with HCC biology and prognosis,^25^, ^26^ but the current cohort is retrospective and single‐centre; prospective multicentre validation will be required before B4GALNT1 can be considered a clinically established predictive marker. Several mechanistic limitations remain. DRM isolation is an operational approximation of lipid‐raft organisation and cannot fully resolve nanoscale membrane domains. GM1 supplementation may have pleiotropic effects, and broader structural or flux‐based studies would strengthen interpretation of the ganglioside axis. The present work also relies heavily on Huh7‐derived resistance models, although directional replication in additional liver‐tumour cells and in xenografts reduces the likelihood of a Huh7‐specific phenomenon. Finally, the single‐cell analysis derives from public datasets and the GAT model depends on predefined genes and graph structure. These limitations define clear priorities for future work, including higher‐resolution membrane imaging, spatial or longitudinal single‐cell approaches, broader patient validation, and expanded testing of upstream mechanisms that drive B4GALNT1 induction during therapy.

## 5 CONCLUSION

This study identifies B4GALNT1 as a functionally relevant regulator of sorafenib resistance in HCC through an integrated analysis spanning public transcriptomic and single‐cell datasets, graph attention network prioritisation, a treatment‐annotated local clinical cohort, multilevel in vitro perturbation, and xenograft validation. The principal findings are as follows. First, B4GALNT1 is upregulated in HCC tumour tissues and in sorafenib‐resistant cells, and higher B4GALNT1 protein expression in a local cohort of 92 sorafenib‐treated patients is associated with poorer treatment response and less favourable survival. Second, B4GALNT1 drives ganglioside metabolic reprogramming along the GM3→GM2→GM1/GD2 axis and increases GM1‐enriched lipid‐raft‐associated membrane fractions, as demonstrated by targeted ganglioside quantification, detergent‐resistant membrane isolation, and orthogonal raft‐disruption/repletion experiments. Third, this membrane remodelling accompanies a composite cell‐death phenotype in which ferroptosis resistance—evidenced by reduced lipid peroxidation, lower labile Fe^2^^+^, higher GPX4/SLC7A11, and ferrostatin‐1‐reversible injury—coexists with enhanced but restricted caspase‐1/GSDMD‐associated signalling characterised by increased cleaved CASP1, GSDMD‐N membrane localisation, and IL‐1β/IL‐18 release without widespread terminal membrane lysis. Fourth, genetic specificity is supported by concordant phenotypes from an independent second‐sgRNA knockout clone, cross‐model validation in HepG2 and MHCC97H backgrounds, and a catalytic‐dead D358A rescue comparison demonstrating that the measured phenotypes depend substantially on B4GALNT1 enzymatic activity. Fifth, exogenous GM1 partially reverses the ferroptosis‐associated and pyroptosis‐related alterations caused by B4GALNT1 deficiency and restores sorafenib tolerance, supporting a functional contribution of ganglioside‐dependent membrane organisation to the resistant state. Sixth, xenograft studies confirm that B4GALNT1 overexpression promotes tumour growth with lower oxidative damage and stronger caspase‐1/GSDMD signalling in vivo, whereas knockout reverses these features and GM1 provides partial rescue.

Collectively, these data support a testable model in which B4GALNT1‐mediated ganglioside remodelling and GM1‐enriched lipid‐raft reorganisation serve as an upstream mechanism that coordinates ferroptosis suppression with restricted pyroptotic membrane permeabilisation, thereby establishing a drug‐adapted state that reduces sorafenib efficacy. However, several limitations should be noted: the clinical cohort is retrospective and single‐centre, the DRM‐based raft isolation is an operational approximation, exogenous GM1 rescue may have pleiotropic effects, and continuous single‐cell fate tracking of permeabilised cells was not performed. Independent prospective clinical validation, higher‐resolution membrane‐domain characterisation, upstream regulatory mechanism elucidation, and expanded model testing therefore remain essential next steps to determine the generalisability and translational potential of the B4GALNT1–ganglioside–lipid‐raft–regulated cell‐death axis in HCC drug resistance.

## AUTHOR CONTRIBUTIONS

L.H.B., B.X. and H.T.S. conceived and designed the study. L.H.B., B.X. and H.T.S. performed the experiments. S.W.R. and X.Y.Y. analysed the data. Z.H.X. and Z.X. wrote the manuscript. All authors reviewed and approved the final version of the manuscript.

## CONFLICT OF INTEREST STATEMENT

The authors declare no conflict of interest.

## ETHICS STATEMENT

All animal experiments were approved by the Animal Ethics Committee of The Seventh People's Hospital Affiliated to Shanghai University of Traditional Chinese Medicine (No. 2025‐AR‐033).

## Supporting information



Supporting Information
Figure S1. TCGA‐LIHC: B4GALNT1 expression, staging associations, survival and diagnostic performance. (A–F) B4GALNT1 expression across tumour/non‐tumour samples and clinicopathological categories in TCGA‐LIHC; (G) Kaplan–Meier overall‐survival analysis; (H) B4GALNT1 expression according to survival status; (I) ROC analysis. Multi‐category comparisons used the Kruskal–Wallis test followed by Dunn's multiple‐comparisons test, and survival curves used the log‐rank test. Statistical significance is denoted as *p < .05, **p < .01, ***p < .001, and ****p < .0001; ns, not significant.

Supporting Information
Figure S2. GSEA and correlation analyses linked to B4GALNT1 expression. (A) Differential‐expression volcano plot comparing B4GALNT1‐high and B4GALNT1‐low tumours; (B–G) GSEA of O‐glycan biosynthesis, ganglio‐series glycosphingolipid biosynthesis, sphingolipid metabolism, ferroptosis, pyroptosis, and glutathione metabolism; (H–K) Spearman's correlations between B4GALNT1 and GSDMD, SLC7A11, CASP1, and GCNT2. Multiple‐testing correction and enrichment criteria are described in Methods.

Supporting Information
Figure S3. Characterisation of sorafenib‐resistant Huh7‐SR line and baseline functional phenotypes. (A, B) B4GALNT1 protein and mRNA measurements in Huh7 and Huh7‐SR cells; (C) JC‐1 analysis of mitochondrial membrane potential; (D) ATP measurement; (E) LDH‐release assay; (F) resistance index; (G) time‐course cell viability under sorafenib. Experiments were performed in three independent biological replicates. Panels A–F were analysed using unpaired two‐tailed Student's *t*‐tests, and panel G was analysed by two‐way ANOVA followed by Sidak's multiple‐comparisons test. Statistical significance is denoted as *p < .05, **p < .01, ***p < .001, and ****p < .0001; ns, not significant.

Supporting Information
Figure S4. Construction and validation of Oe‐B4GALNT1 and KO‐B4GALNT1 cell models. (A–F) Construction and validation of Oe‐B4GALNT1 and KO‐B4GALNT1 cell models, including protein and mRNA measurements, immunofluorescence, cell morphology, and basal proliferation. Experiments were performed in three independent biological replicates. Quantitative multi‐group comparisons were analysed by one‐way ANOVA followed by Tukey's multiple‐comparisons test. Statistical significance is denoted as *p < .05, **p < .01, ***p < .001, and ****p < .0001; ns, not significant.

Supporting Information
Figure S5. B4GALNT1 drives GM2/GM1 accumulation and lipid‐raft remodelling; orthogonal raft perturbations strengthen specificity. (A) TLC analysis of GM3, GM2, and GM1; (B) TLC‐band quantification; (C, D) GM2 immunofluorescence and quantification; (E, F) GM1 immunofluorescence and quantification; (G) targeted measurement of GM3, GM2, GM1, and GD2; (H) GM1 enrichment in DRM fractions; (I) MβCD treatment (10 mM, 30 min); (J) cholesterol repletion (.5 mM) after MβCD treatment; (K) relative DRM enrichment of Flotillin‐1, Caveolin‐1, and CTxB‐detected GM1; (L) PI‐positive cells after vehicle or erastin (5 µM, 24 h); (M) PI‐positive cells after control, erastin, or erastin + ferrostatin‐1 (1 µM). Experiments were performed in three independent biological replicates. Panels A–H were analysed by one‐way ANOVA followed by Tukey's multiple‐comparisons test; grouped treatment comparisons in panels I–M were analysed by two‐way ANOVA followed by Sidak's multiple‐comparisons test. Statistical significance is denoted as *p < .05, **p < .01, ***p < .001, and ****p < .0001; ns, not significant.

Supporting Information
Figure S6. GM1 supplementation restores sorafenib tolerance in B4GALNT1‐deficient cells. (A–E) CCK‐8 viability, Annexin V/PI analysis, sorafenib dose–response/IC_50_ analysis, and colony formation in KO‐B4GALNT1 + Sorafenib and KO‐B4GALNT1 + GM1 + Sorafenib groups; (F) cellular GSH content. Experiments were performed in three independent biological replicates. Two‐group endpoint comparisons were analysed using unpaired two‐tailed Student's *t*‐tests, and dose–response curves were fitted by nonlinear regression. Statistical significance is denoted as *p < .05, **p < .01, ***p < .001, and ****p < .0001; ns, not significant.

Supporting Information
Figure S7. Cross‐model validation of the B4GALNT1–ganglioside–raft axis in additional liver‐tumour cell models. (A–E) Cross‐model validation in HepG2 and MHCC97H parental and sorafenib‐resistant derivatives, including B4GALNT1 expression, sorafenib response, ferroptosis‐associated readouts, and caspase‐1/GSDMD‐related measurements. Experiments were performed in three independent biological replicates. Dose–response curves were fitted by nonlinear regression; grouped endpoint comparisons were analysed by two‐way ANOVA followed by Sidak's multiple‐comparisons test. Statistical significance is denoted as *p < .05, **p < .01, ***p < .001, and ****p < .0001; ns, not significant.

Supporting Information
Figure S8. Independent second‐sgRNA knockout validation in Huh7 and Huh7‐SR cells. (A) Western blot analysis of B4GALNT1, GPX4, and SLC7A11 in WT, sgNC, and KO‐2 cells from Huh7 and Huh7‐SR backgrounds; (B) GPX4 and SLC7A11 RT‐qPCR; (C) Western blot analysis of cleaved CASP1 and GSDMD‐N; (D) ELISA measurement of IL‐1β and IL‐18; (E) 72‐h sorafenib dose–response curves; (F) MDA assay; (G, H) DCF‐DA imaging and quantification; (I) PI‐positive‐cell quantification; (J) MitoSOX imaging. Experiments were performed in three independent biological replicates. Grouped endpoint data were analysed by two‐way ANOVA followed by Sidak's multiple‐comparisons test; in panel E, dose–response curves were fitted by nonlinear regression. Scale bars are indicated in the fluorescence images. Statistical significance is denoted as *p < .05, **p < .01, ***p < .001, and ****p < .0001; ns, not significant.

Supporting Information
Figure S9. Time‐resolved membrane permeabilisation, caspase dependence, and ferrostatin‐1 rescue in Huh7‐SR cells. (A) Time‐resolved PI‐positive and YO‐PRO‐1‐positive cell fractions; (B) LDH‐release kinetics; (C) cleaved caspase‐3 fluorescence intensity; (D) YO‐PRO‐1‐positive cells after DMSO, VX‐765 (10 µM), Ac‐YVAD‐cmk (20 µM), Z‐DEVD‐fmk (20 µM), or Z‐VAD‐fmk (20 µM); (E) lipid ROS in ferrostatin‐1 rescue modules; (F) cell‐viability time courses; (G) ATP time courses; (H) LDH‐release time courses; (I) time to the first detectable YO‐PRO‐1‐positive event; (J) YO‐PRO‐1‐positive cells remaining viable at 24 h. Experiments were performed in Huh7‐SR cells with three independent biological replicates. Time‐course data used two‐way ANOVA with Sidak's multiple‐comparisons test, and endpoint data used one‐way ANOVA with Tukey's multiple‐comparisons test. Statistical significance is denoted as *p < .05, **p < .01, ***p < .001, and ****p < .0001; ns, not significant.

Supporting Information
Figure S10. Wild‐type B4GALNT1 rescue, but not B4GALNT1‐D358A rescue, restores B4GALNT1‐associated phenotypes in the KO background. (A) Relative GM1 enrichment; (B) relative GPX4 and SLC7A11 protein expression; (C) relative cleaved CASP1 and GSDMD‐N protein expression; (D) relative sorafenib IC_50_ in KO + Empty vector, KO + WT‐B4GALNT1 rescue, and KO + B4GALNT1‐D358A rescue groups. Values are expressed relative to the KO + Empty vector reference. Experiments were performed in three independent biological replicates. Panels A and D used one‐way ANOVA with Tukey's multiple‐comparisons test; panels B and C used two‐way ANOVA with Sidak's multiple‐comparisons test. Data are presented as mean ± SD. Statistical significance is denoted as *p < .05, **p < .01, ***p < .001, and ****p < .0001; ns, not significant.

## Data Availability

All data generated or analysed during this study are included in this article and/or its supplementary material files. Further inquiries can be directed to the corresponding author.
